# Brain-wide connections of the parvicellular subdivision of the basolateral and basomedial amygdaloid nuclei in the rats

**DOI:** 10.3389/fncir.2025.1575232

**Published:** 2025-04-25

**Authors:** Ge Zhu, Sheng-Qiang Chen, Run-Zhe Ma, Hui-Ru Cai, Jin-Yuan Zhang, Yi-Min Peng, Dian Lian, Song-Lin Ding

**Affiliations:** ^1^Key Laboratory of Neuroscience, School of Basic Medical Science, Guangzhou Medical University, Guangzhou, China; ^2^Department of Psychology, School of Health Management, Guangzhou Medical University, Guangzhou, China; ^3^Allen Institute for Brain Science, Seattle, WA, United States

**Keywords:** connectivity, ventral hippocampus, hypothalamus, entorhinal cortex, nucleus accumbens, amygdala, midline thalamus, cadherin 13

## Abstract

As the core area of emotion regulation, the amygdala is involved in and regulates many related behaviors, such as fear, anxiety, depression, as well as reward, learning, and memory. Most previous connectional studies have focused on the anterior and middle parts of the basolateral nucleus (BL) and basomedial nucleus (BM) of the amygdala. Little is known about the brain-wide connections of the posterior part of the BL and BM (termed parvicellular subdivision of the BL and BM, i.e., BLpc and BMpc). In this study, brain-wide afferent and efferent projections of the BLpc and BMpc in the rats are investigated using both retrograde and anterograde tracing methods. Both common and differential connections of the BLpc and BMpc are revealed. Major common inputs of both regions originate from the ventral hippocampal CA1 and prosubiculum, sublenticular extended amygdala, anterior basomedial nucleus, midline thalamic nuclei, endopiriform nucleus, dorsal raphe, piriform cortex and lateral entorhinal cortex. The BLpc receives preferential inputs from agranular insular cortex, amygdalopiriform transition area, periaqueductal gray, parataenial nucleus and anterior cortical nucleus of the amygdala. The BMpc preferentially receives its inputs from the peripeduncular nucleus, paraventricular nucleus of thalamus, ventromedial hypothalamic nucleus (VMH), caudal bed nucleus of stria terminalis (BST), medial amygdaloid nucleus and posterior cortical nucleus of the amygdala. Major differential outputs of the BLpc and BMpc are also obvious. The BLpc projects mainly to nucleus accumbens, rostral BST, lateral central amygdaloid nucleus (Ce), intermediate BL and BM. The BMpc sends its main outputs to VMH, medial Ce, caudal BST, prosubiculum, and perirhinal-ectorhinal cortices. These major findings are further confirmed with anterograde viral tracing in mice. Compared with previous findings in monkeys, our findings in rodents suggest that the BLpc and BMpc have overall similar connectional patterns across species. In addition, some gene markers for BM subdivisions are identified. All these findings would provide an important anatomical basis for the understanding of emotion-related neuronal circuits and diseases and for cross-species comparison of the subcircuits in amygdaloid complex.

## 1 Introduction

The amygdala is a heterogeneous brain region that processes and regulates many aspects of emotion such as fear, anger, pleasure, anxiety, reward and related memory. The amygdala can be subdivided into several groups with the basolateral nuclear group at the center (De Olmos et al., 1985; Price et al., 1987; Pitkanen, 2000). The basolateral nuclear group contains three major nuclei, and these include lateral (La), basolateral (BL) (or basal [B]) and basomedial (BM) (or accessory basal [AB]) nuclei depending on different nomenclature (e.g., De Olmos et al., 1985; Amaral et al., 1992; Pitkanen, 2000; Fudge et al., 2002; Paxinos and Watson, 2007; Bakken et al., 2016).

In human and non-human primate, the BL (or B) is further parcellated into magnocellular (Bmc), intermediate (Bi) and parvicellular (Bpc) subdivisions (Amaral et al., 1992; Fudge et al., 2002; Bakken et al., 2016; Mchale et al., 2022) or into dorsal (BLD), intermediate (BLi) and ventral (BLV) subdivisions, respectively (e.g., Paxinos et al., 2009; Ding et al., 2016; Ding et al., 2022). For consistent use of these terminology, we use BLmc, BLi and BLpc in this study. In rodents, however, parcellation of the BL is inconsistent among different research groups. For example, the BL in rats and mice is often subdivided into the anterior and posterior parts (BLA and BLP, respectively) by many authors (De Olmos et al., 1985; Swanson, 1992; Paxinos and Watson, 2007; Wang et al., 2020). Other authors use the terms Bmc (BLmc), Bi (BLi) and Bpc (BLpc) for subdivisions of the rodent BL (or B) along the anterior to posterior axis (Price et al., 1987; Savander et al., 1995; Pitkanen, 2000). These two segmentation schemes for rodents only partially overlap with each other (e.g., Savander et al., 1995 versus Paxinos and Watson, 2007) and roughly the BLP contains the BLi and BLpc. Although the boundaries between these BL subdivisions are variably placed in rodent literature, we use the same terms BLmc, BLi and BLpc as in monkeys to facilitate comparison of their connectivity across these species.

Comparison of monkey and rodent BL indicates that monkey BLmc, BLi and BLpc display strong, intermediate and weaker AChE staining at dorsal, intermediate and ventral levels of the BL, respectively (Price et al., 1987; Fudge et al., 2002; Paxinos et al., 2009). Additionally, the overall cell sizes in monkey BL decrease from dorsal to ventral levels of the BL (Amaral et al., 1992; Paxinos et al., 2009). In contrast, rodent BL shows strong, intermediate and weaker AChE staining along the anterior to posterior axis of the BL (Paxinos and Watson, 2007). Consistently, the overall cell sizes in rodent BL decrease gradually from anterior to posterior levels (Paxinos and Watson, 2007). This comparison suggests that rodent BLpc is probably in the most posterior part of the BL.

As for the subdivisions of the BM (or AB) in monkeys, the BM (or AB) is subdivided dorsoventrally into BMD (or ABmc, with mostly large cells) and BMV (or ABpc; with mostly smaller cells) with an additional ABvm included sometimes (Amaral et al., 1992; Fudge et al., 2002; Paxinos et al., 2009). Here we mainly use the terms BMmc and BMpc. Interestingly, in rodents, the BM is subdivided into anterior (BMA, with mostly smaller cells) and posterior (BMP, with mostly larger cells) parts (De Olmos et al., 1985; Swanson, 1992; Paxinos and Watson, 2007). Although BMA in rodents was sometimes treated as the possible equivalent of the monkey BMpc, major differential connections of the BMA in rodents and the BMpc in monkeys do not support this claim (e.g., Amaral et al., 1992; Petrovich et al., 1996; Pitkanen, 2000; Decampo and Fudge, 2013; Mchale et al., 2022). Since the rodent BMP contains overall larger cells in its anterior part and smaller cells in its posterior part, we hypothesize that the posterior part of the BMP likely corresponds to the BMpc in monkeys. In this study, we treat the anterior and posterior parts of the rodent BMP as the BMmc and BMpc, respectively. Therefore, both the BLpc and BMpc are probably in the most posterior part of the BL and BM.

There are two additional approaches to test our hypothesis about the localization of the BLpc and BMpc, in addition to their orientation and AChE staining intensity mentioned above. The first is to compare the connectivity between the BLpc and adjacent BLi as well as between the BMpc and adjoining BMmc. Obvious differences in the connectivity between the two pairs would support a more accurate subdivision of the BLpc or BMpc. The second is to compare the connectivity of BLpc or BMpc in rodents with that reported in monkeys. Similar main connectivity would support homologous subdivisions across these species.

Since most of previous rodent studies focus on the BLmc-BLi and/or treat the BMP as a single entity, very limited connectional data is available for the BLpc and BMpc in rodents. Therefore, the main purpose of the present study is to reveal brain-wide connections of the strictly defined BLpc and BMpc in rats and then compare the connections of these two subdivisions with those reported in monkeys. Overall, the connectional patterns of the BLpc and BMpc revealed in this study, together with their orientation and the staining intensity of AChE and other markers, support the conclusion that the BLpc and BMpc defined in the present study are probably the equivalents of those in monkeys.

## 2 Materials and methods

### 2.1 Animals

This study used 40 adult Sprague–Dawley rats (Beijing Vitare Laboratory Animal Technology Co., Ltd., Beijing, China) weighing 250 g and 430 g, with a 50/50 split between females and males. All rats were housed in the same room at 22–25 degrees Celsius, grown in a pre-established 12-h alternating darkness environment, and kept in rat cages with free access to food and water. All experiments were conducted under deep anesthesia to reduce the pain of the animals. All experimental procedures were performed by the documents approved by the Laboratory Animal Management and Use Committee of Guangzhou Medical University.

### 2.2 Brain stereotactic surgery and tracer injection procedure

The methods for brain stereotactic surgery have been described in our previous studies (e.g., Chen et al., 2021; Xiang et al., 2023). Firstly, the SD rats were weighed, and sodium pentobarbital (40 mg/kg) was injected intraperitoneally. Surgery started after all limb reflexes disappeared, corneal reflexes disappeared, and a state of deep anesthesia was entered. The head of the rat was fixed on a brain stereotaxic apparatus (Shenzhen Reward Technology Company), and a small opening of about 2 cm was made at the top of the head to fully expose the skull. By adjusting the adapter and ear rods to make sure the bregma and lambda points are on the same horizontal line, the coordinate point positions of the target brain regions were determined according to the rat brain atlas (Paxinos and Watson, 2007). A small window was opened with a cranial brick over the target brain regions of tracer injections. The target brain regions include the BLpc, BMpc and ventromedial hypothalamic nucleus (VMH). The tracers used in the present study include a retrograde tracer Fluoro-Gold (FG; Fluorochrome) and a bidirectional tracer biotinylated dextran amine (BDA, Invitrogen). 0.04 μL of 4% FG or 0.05 μL of 10% BDA was injected into each of the target regions using a 1 μL Hamilton syringe (Thermo Fisher Science, Waltham, MA, USA). The injection needle was left in place for 10 min and then withdrawn slowly to prevent tracer leakage. Sterilization and wound closure were then performed. Finally, the postoperative rats were placed in a warm box for awakening, and when they were fully awakened, they were returned to their cages and given adequate food and water.

### 2.3 Processing of brain tissues

After 7–9 days postoperatively, the rat was injected intraperitoneally with sodium pentobarbital (60 mg/kg), and after deep anesthesia, the limbs of the rat were fixed on a perfusion board, and saline (0.9%) was used to perfuse the rat through the aorta of the heart. Then 4% paraformaldehyde solution (in 0.1 M PBS, pH7.6) was used to perfuse the rat. After the perfusion was completed, the brain was carefully extracted and put into 4% of the paraformaldehyde solution. After 24 h, the brain was first placed in 15% sucrose (in 0.05 M PBS, pH 7.3). After the brain sunk to the bottom of the container, it was transferred to 30% sucrose until the brain bottomed (about 2–3 days). The whole brain was cut with a frozen sectioning machine (Leica, Germany) and serial coronal sections (40 μm) were collected in sequences of five sets. The collected brain sections were subjected to subsequent histochemical staining.

### 2.4 Nissl staining

Selected brain sections for Nissl staining were picked out, washed three times with 0.05 M PBS (pH7.3), and then mounted and dried in a drying box for 48 h. The sections were placed in distilled water for 1 min, then put in Niehl’s staining solution (Shanghai Shengong Biological Engineering Co., Ltd.) for 30 min, and then washed in distilled water for 3 min. The sections were placed in 95 and 100% alcohol for 1 min each and then put in biological agent TO for transparency. Finally, the sections were sealed with neutral resin and dried in a drying oven.

### 2.5 Histochemical staining for FG and BDA labeling

After examining the location and extent of FG injection using an inverted fluorescence microscope (ZEISS), a set of sections from each brain were processed for histochemistry of FG and BDA. Firstly, the sections were washed three times in 0.05 M PBS, after which they were placed in 3% hydrogen peroxide, washed again with PBS buffer, and then closed in 5% bovine serum protein for 1 h, followed by placing the sections in 0.3% Triton-100 and primary antibody (mouse anti-FG, AB153-I, 1:10,000, Sigma-Aldrich, St. Louis, MO. United States) in a refrigerator at 4 degrees Celsius overnight. The sections were then placed in secondary antibody (biotin-labeled goat anti-mouse/rabbit immunoglobulin, Boster BioTechnology, Pleasanton, CA, United States), followed by incubation in streptavidin-biotin complex (SABC kit, Boster BioTechnology) for 60 min, and finally, the sections were incubated with 0.05% diamino Benzidine (DAB) for color development and then terminated with 0.01 M PBS. Finally, the sections were mounted, dried and put in gradient alcohol and the biotin TO before they were sealed with neutral resin.

The brain sections from the cases with BDA injections were taken out, washed with 0.05 M PBS, and then placed in 0.3% Triton to break the membrane for 90 min. Then, the slices were incubated with Streptavidin-Biotin-Peroxidase Complex SABC for 3 h. After being washed with 0.01 M PBS, the sections were used to develop the color with 0.05% diaminobenzidine (DAB) and then terminated with 0.01 M PBS. The staining was then terminated with 0.01 M PBS. Finally, the sections were mounted, dried and put in gradient alcohol and the biotin TO and then sealed with neutral resin.

### 2.6 Image acquisition and processing

We used an inverted fluorescence microscope (ZEISS) (Axio Observer3) to observe and capture the fluorescent images of the injection sites and labeled neurons. Sections that underwent BDA histochemical staining were digitized using a tissue section scanner (Aperio CS2, Leica) for labeled neurons and axon terminals. The images obtained above were processed in Photoshop 2023 for brightness, contrast, and size adjustment. To clearly show the brain-wide distribution of the neuronal and axon terminal labeling following injections of FG and BDA, we projected labeled neurons and/or axon terminals to the corresponding atlas plates of the rat brain atlas (Paxinos and Watson, 2007). The density of retrogradely labeled neuronal cell bodies (FG) and anterogradely labeled axon terminals (BDA) were plotted separately.

### 2.7 Evaluation of FG and BDA injection sites

Among the 40 rats used in this study, those with injection sites largely missing their targets (*n* = 10) were excluded from further analysis. In the remaining 30 cases, 10 cases have the FG or BDA injections in the BLpc, 12 cases in the BMpc, and 8 cases in other connectionally related brain regions (e.g., VMH and PIL). The injection sites from 12 cases are shown in Supplementary Figure 1. Overall, the injection cores are centered in their target regions (indicated by #) although some variabilities exist in their size, location and intensity. In general, the FG injection sites appear larger than the BDA injections sites mainly due to the strong FG signals surrounding the injection cores under fluorescent microscope.

### 2.8 Semi-quantitative or quantitative analysis of FG-labeled neurons and BDA-labeled axon terminals

To evaluate the relative density of FG-labeled neurons or BDA-labeled terminals following the BLpc and BMpc injections, we semi-quantitatively scored the densities of the labeled neurons or terminals. Twelve cases with relatively accurate FG (*n* = 6) or BDA (*n* = 6) injections were selected (3 cases for each group), and the evaluation was performed on the roughly corresponding sections between the two groups (BLpc and BMpc). The semi-quantitative densities of FG-labeled neurons or BDA-labeled axon terminals are grouped into none, low, moderate, and dense. To further confirm our main findings of the differential inputs to the BLpc and BMpc, FG-labeled neurons in the brain regions with potential difference between the two groups (*n* = 3 each group) were counted on matched sections and analyzed using Image J. Based on the results of semi-quantitative evaluation, FG-labeled neurons in the following brain regions were counted, and these include BSTc, VMH, PT, BMA, MeP and APir. The average number (mean) and sum of the labeled neurons counted from three cases of each group were taken for graphical analysis in GraphPad Prism 10. Two-independent sample t-tests was used for the analysis of significant difference between the two groups.

### 2.9 Nomenclature of amygdaloid subdivisions

As mentioned in the Introduction section, different terminologies are used for amygdaloid divisions and subdivisions in literature. In the present study, the major divisions are termed as central (Ce), medial (Me), cortical (Co), lateral (La), basolateral (BL), basomedial (BM) amygdaloid nuclei and amygdalohippocampal area (AHi) following the terminologies of Price et al. (1987) and Paxinos and Watson (2007). For subdivisions of the BL, however, the terms magnocellular, intermediate and parvocellular subdivisions were adopted to segment the BL (i.e., BLmc, BLi and BLpc, respectively), modified from the terms Bmc, Bi and Bpc of Pikkarainen and Pitkänen (2001). As for subdivisions of the BM, the terms anterior subdivision (BMA, mostly small cells) and posterior subdivision (BMP; mostly larger cells) of the BM are used following the terms of Swanson (1992) and Paxinos and Watson (2007). Since the BMP has mostly large and small cells in its anterior and posterior parts, respectively, we further subdivide the BMP into the BMmc (anterior part) and BMpc (posterior part). Thus, we have incorporated a few existing parcellation schemes in the present study to harmonize the divisions and subdivisions of the amygdala and to facilitate comparison between rodent and primate amygdala. It should also be pointed out that the overall cell sizes in the BLpc are larger than those in the BMpc, making the BLpc and BMpc distinguishable. The terminology for other brain structures used in the present studies are mostly based on Paxinos and Watson (2007) with an exception for the differential subiculum (SB) and prosubiculum (ProS), which are derived from a recent comprehensive study by Ding et al. (2020), and for the adoption of periamygdaloid cortex (PAC), which is derived from Price et al. (1987) and Amaral et al. (1992). The PAC roughly corresponds to the posterolateral cortical nucleus (PLCo) of Paxinos and Watson (2007). Accordingly, the posteromedial cortical nucleus (PMCo) is modified as the posterior cortical nucleus (PCo), which is paired with the anterior cortical nucleus (ACo).

## 3 Results

### 3.1 Location, topography, and cytoarchitecture of the BLpc and BMpc

**Figure 1:**
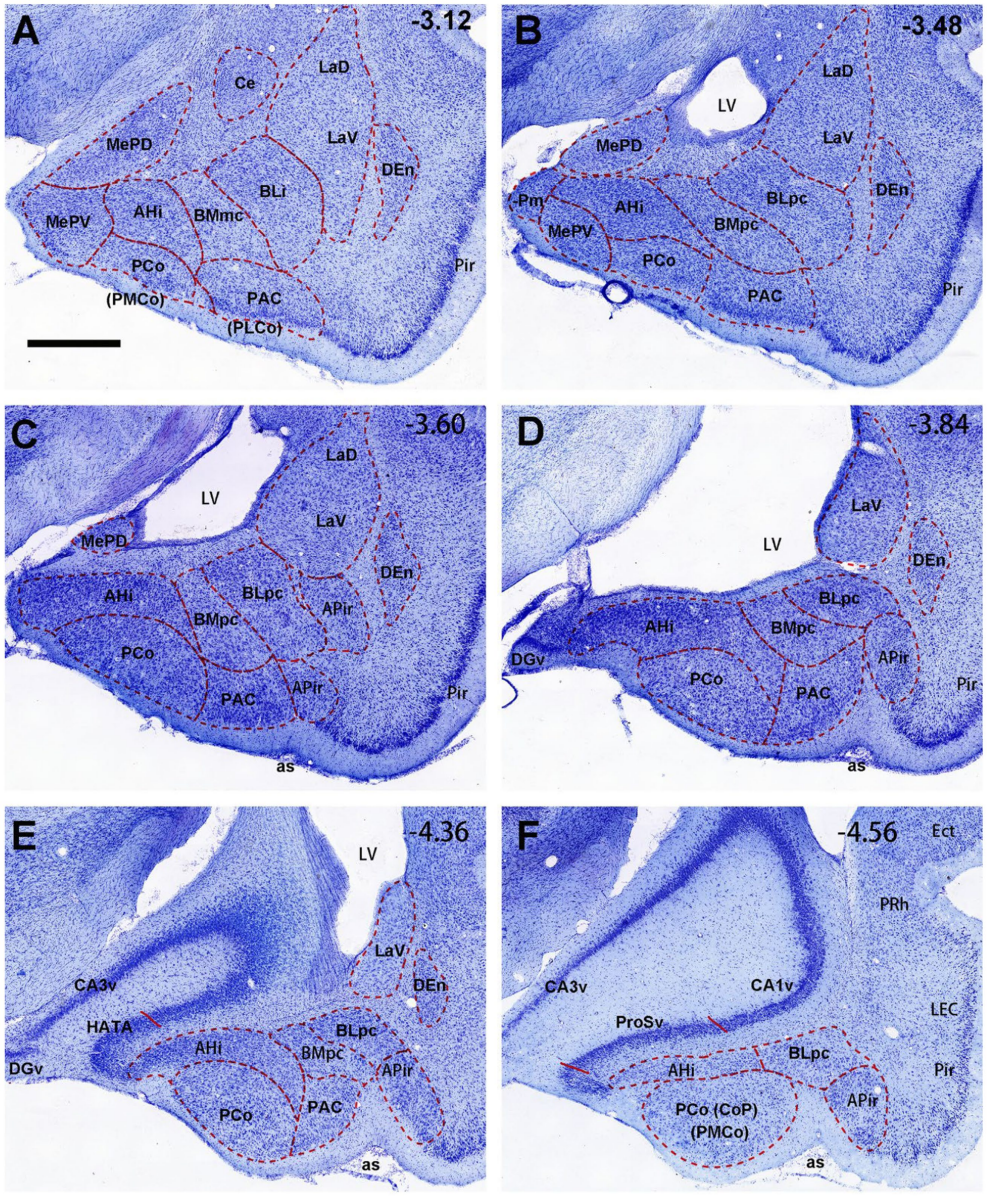
General location, topography and cytoarchitecture of the BLpc and BMpc in rats. **(A–F)** Sequential Nissl-stained coronal sections from the posterior portion of the amygdala in rats. The anterior–posterior coordinates are indicated in the upper right corner of each image. Note that BLpc and BMpc start at about the level where lateral ventricle (LV) appears ventrally and adjoins the amygdala **(B)**. For abbreviations see the list. Scale bar: 800 μm in **(A)** for **(A–F)**.

**Figure 2:**
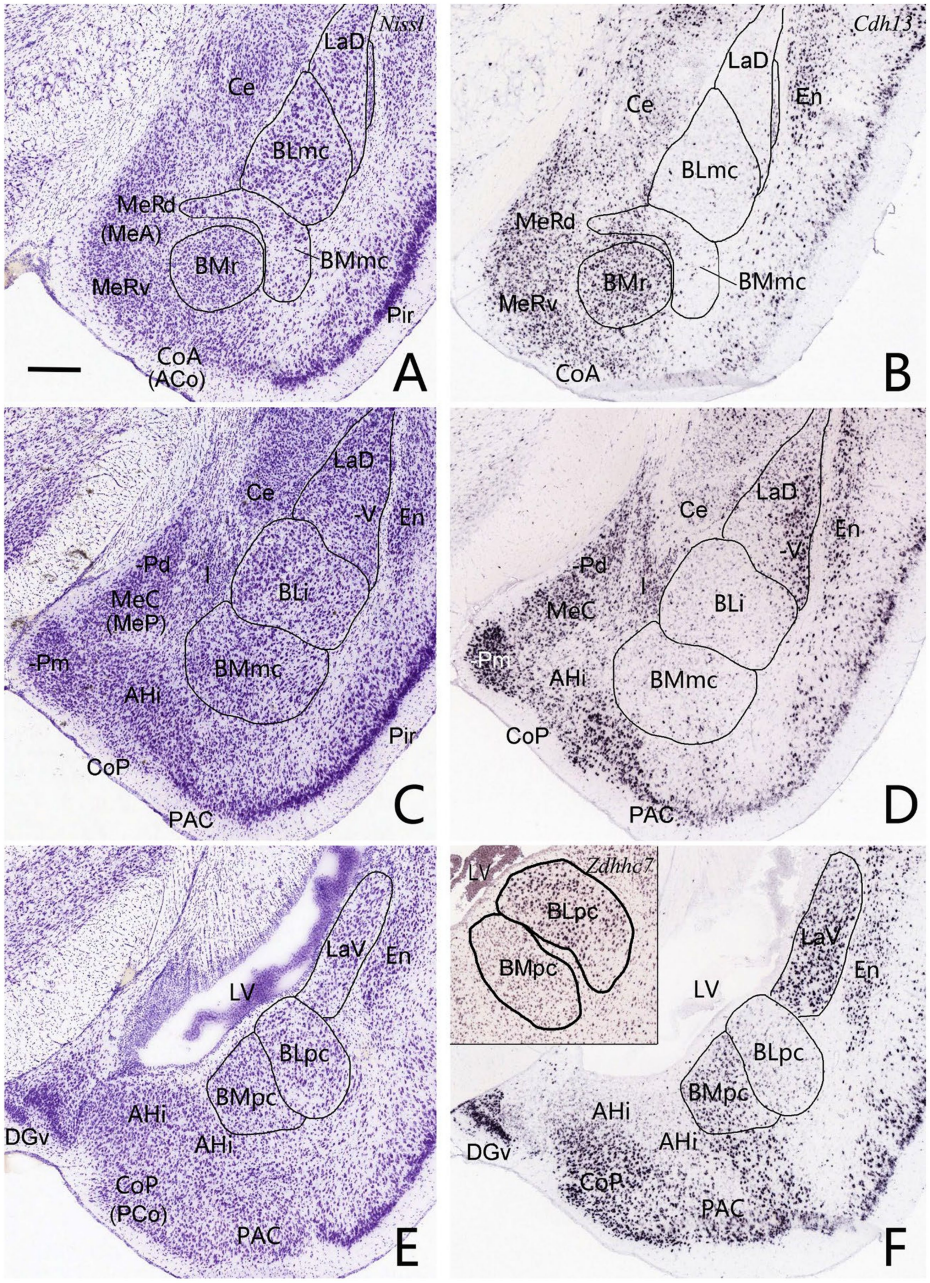
Location, topography, cytoarchitecture and gene markers of the BLpc and BMpc in mice. **(A,C,E)** Three sequential Nissl-stained coronal sections showing the location, topography, and cytoarchitecture of the BL and BM subdivisions. **(B,D,F)** Three adjacent sections to the Nissl-stained sections showing *Cdh13* expression in the amygdaloid subdivisions. Note that *Cdh13* is strongly expressed in the BMr and BMpc but not in the BMmc. In addition, LaV but not LaD displays strong *Cdh13* expression. (Inset in **F**) Strong *Zdhhc7* expression in the BLpc but not the BMpc. Thus, both *Cdh13* and *Zdhhc7* can be used to delineate the BLpc and BMpc. For abbreviations see the list. Scale bar: 300 μm in **(A)** for all panels.

The overall locations, topography and cytoarchitecture of the BLpc, BMpc and other adjoining amygdaloid regions in the rats have been described in literature (e.g., Price et al., 1987; Savander et al., 1995; Petrovich et al., 1996; Pikkarainen and Pitkänen, 2001). Briefly, as shown in sequential coronal sections of Figure 1, the BLpc is located dorsolateral to the BMpc, ventromedial to the La and posterior to the BLi (Figures 1A–D). At the ventral aspect, the BLpc adjoins amygdalopiriform transition cortex (APir), the BMpc abuts with the PAC (PLCo), and the AHi adjoins the PCo (PMCo) and MePD (Figures 1A–F). Histologically, cells in the BLpc are densely packed while those in the BMpc are less densely packed (Figures 1A–D). Additionally, cells in the BLpc are mostly larger than those in the BMpc. Based on overall changes of cell sizes in the BL and BM along the anterior–posterior axis, we find that both BLpc and BMpc exist posterior to the level where the ventral part of lateral ventricle (LV) appears (Figure 1B). Slightly anterior to this level is a transition region where many larger and some small cells co-exist (Figure 1A). It is also important to mention that the whole BL (including the BLi and BLpc) shows overall stronger AChE staining compared to the whole BM (including the BMmc and BMpc) and La (LaD and LaV). Both the BM and La display much weaker AChE staining than the BL (e.g., Price et al., 1987; Paxinos and Watson, 2007), making them distinguishable from the BL. Finally, our further subdivision of the BMP into BMmc and BMpc also receives support from mouse gene expression data. For example, the gene *Cdh13* (*cadherin 13*) is strongly expressed in the BMA (BMr) and BMpc but not in the BMmc (Figures 2A–F). Other genes such as *Chrm1* and *Chrm 2* (*cholinergic receptor muscarine 1 and 2*, respectively) are expressed in the BMmc but not in the BMpc and BMr (see *Chrm2* in Supplementary Figures 2A,B). It is also noted that the gene *Zdhhc7* (*zinc finger, DHHC domain containing 7*) is strongly expressed in all BL subdivisions (with a gradient) but not in BM subdivisions (see Supplementary Figure 2C and the inset in Figure 2F).

### 3.2 Brain-wide afferent connections of the BLpc

**Figure 3:**
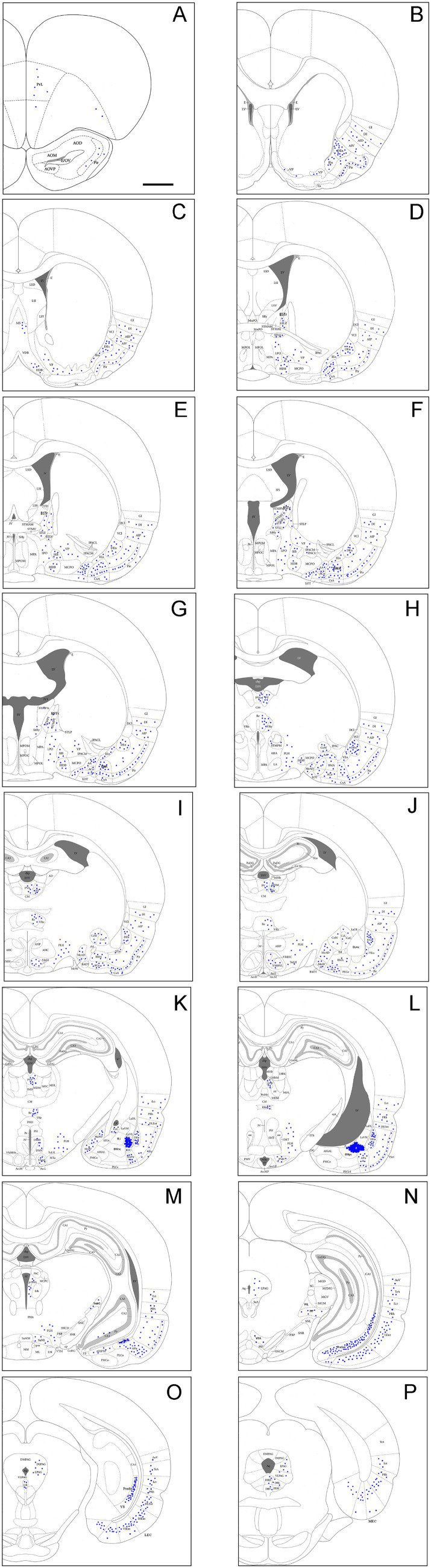
Brain-wide afferent projections of the BLpc in the rats. **(A–P)** Schematic illustration of FG-labeled neurons in sequential anterior **(A)** to posterior **(P)** coronal sections following an FG injection into the BLpc. The injection site (#) is shown as the crowded dot region in panel L. See text for explanation. For abbreviations see the list. Scale bar: 2 mm in **(A)** (apply to all panels).

**Figure 4:**
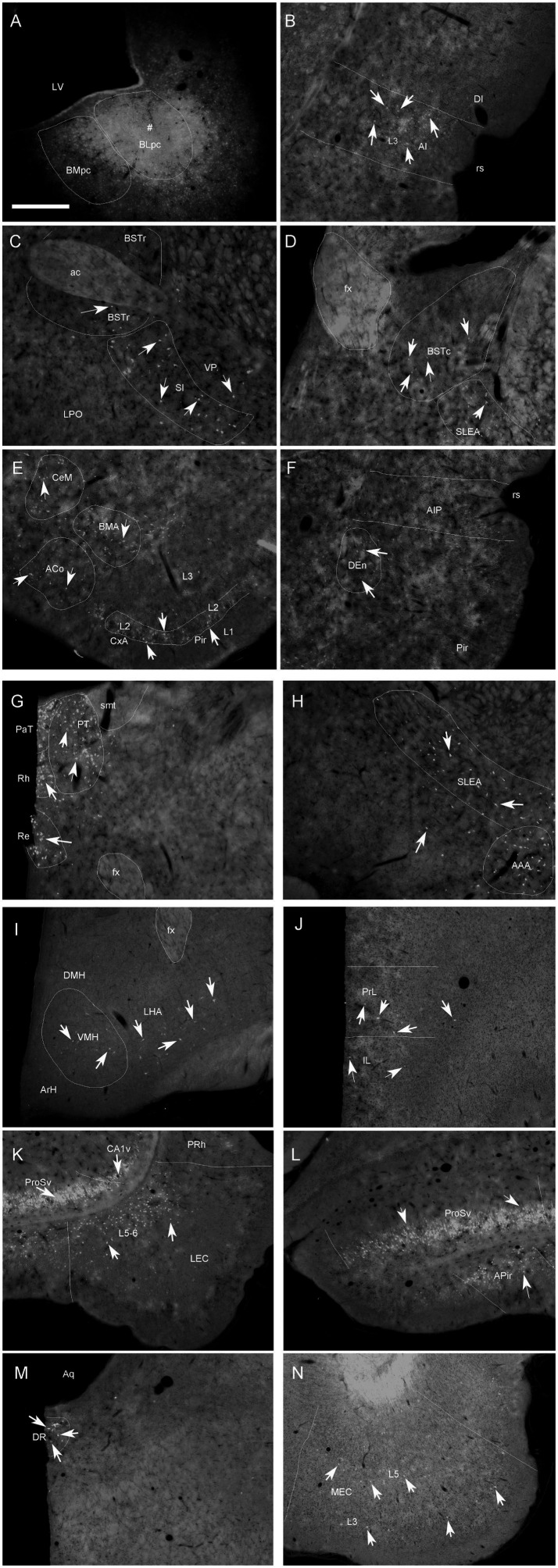
Representative brain regions that send afferent projections to the BLpc in the rat. **(A)** One FG injection site (#) restricted in the BLpc (see the ventral LV). **(B–N)** FG-labeled neurons in the AI **(B)**, BSTr, VP/SI **(C)**, BSTc, SLEA **(D)**, ACo, BMA, CxA, Pir **(E)**, DEn, AIP **(F)**, PaT, PT, Rh, Re **(G)**, SELA, AAA **(H)**, VMH, LHA **(I)**, PrL **(J)**, ProSv, CA1v, LEC **(K)**, APir **(L)**, DR **(M)** and MEC **(N)**. Arrows indicate some labeled neurons. For abbreviations see the list. Scale bar: 500 μm in **(A)** (applies to all images).

To evaluate brain-wide afferent connections of the BLpc, we successfully performed FG injections into the BLpc in four rats and examined the distribution of retrogradely labeled neurons in the whole brain. Figures 3A–P shows one representative case with an FG injection restricted in the BLpc (see injection site # in Figures 3L,4A). Overall, FG-labeled neurons are mainly found in the ventral part of the brain hemisphere (Figures 3A–P).

Specifically, a moderate number of FG-labeled cells are observed in the piriform cortex (Pir), cortical transition area (CxA) (Figures 3A–M) and dorsal endopiriform nucleus (DEn) (Figures 3B–L, 4F). Some FG-labeled neurons are also seen in the basal forebrain regions including medial septal nucleus (MS),vertical and horizontal parts of the nucleus of diagonal band (VDB and HDB), and ventral pallidum/substantia inonminata (VP/SI) (Figures 3C–F, 4C), and in the hypothalamic regions including lateral preoptic areas (LPO) (Figures 3D–G), lateral hypothalamic area (LHA or PLH) (Figures 3H–M, 4I), ventromedial hypothalamic nucleus (VMH) (Figures 3I–K, 4I), dorsomedial hypothalamic nucleus (DMH), ventral premammillary nucleus (PMv) and supramammillary nucleus (SUM) (Figures 3K–M).

In the amygdaloid complex, a moderate number of FG-labeled neurons are seen in the anterior regions of the amygdala including the anterior amygdaloid area (AA or AAA), anterior cortical nucleus (ACo) and anterior or rostral division of the BM (BMA or BMr) (Figures 3E–J, 4E, H). In the medial amygdaloid nucleus (Me), the anterior (MeAD and MeAV) and posterior (MePD and MePV) subdivisions of the Me contain fewer and more FG-labeled neurons, respectively (Figures 3H–K). In addition, the posteromedial part of AHi (AHiPM) contains sparsely labeled neurons (Figure 3M). Some FG-labeled neurons are also scattered in the medial (CeM), capsular (CeC) and lateral (CeL) subdivisions of the central amygdaloid nucleus (Ce) (Figures 3H–K). Finally, many labeled neurons are found in the amygdalopiriform transition area (APir, Figures 3K–N, 4L).

In the extended amygdala (EA) or bed nucleus of the stria terminalis (ST or BST), FG-labeled neurons are sparsely distributed in the rostral (BSTr; Figures 3D,E) and caudal (BSTc; Figures 3F,G) subdivisions of the BST. However, more labeled neurons are found in the sublenticular EA (SLEA; Figures 4D,H). The BSTr is located dorsal and ventral to the anterior commissure (ac), and between the septal nuclei and internal capsule (ic) (Figures 3D,E). The BSTc lies caudal to the anterior commissure and rostral to the anterior thalamic nucleus (Figures 3F,G). SLEA is located ventral to the VP (Figure 4H).

In the thalamus, many FG-labeled neurons are detected in the paraventricular nucleus of the thalamus (PaT or PV), parataenial nucleus (PT) (Figures 3H–L, 4G), rhomboid thalamic nucleus (Rh) and nucleus reuniens (Re) (Figures 3H–L, 4G). Some labeled neurons are also seen in the subgeniculate nucleus (SubG; Figure 3M) and peripedundcular nucleus (PP; Figure 3N). The PP is defined based on our recent findings (Cai et al., 2024).

In the brainstem, scattered FG-labeled neurons are observed in the periaqueductal gray (PAG; Figures 3M–P), ventral tegmental area (VTA, Figure 3N) and dorsal raphe (DR; Figures 3O,P, 4M).

In hippocampal regions, extremely densely labeled neurons are seen in the ventral prosubiculum (ProSv) (Figures 3N,O, 4K,L). Densely labeled neurons are also observed in ventral CA1 (CA1v, Figures 3N, 4K). In addition, many FG-labeled cells are detected in the deep layers (L5-6) of the lateral entorhinal cortex (LEC; including at least the DLEnt, DIEnt and VIEnt in the atlas) (Figures 3N,O, 4K), with much fewer labeled neurons in the medial entorhinal cortex (MEC, Figures 3P, 4N).

In the neocortical regions, sparse cell labeling is detected in the dysgranular insular cortex (DI) with slightly more labeled neurons seen in the agranular insular cortex (AI; including AID, AIV and AIP) (Figures 3B–J, 4B,F). Fewer FG-labeled neurons are detected in the prefrontal cortex, such as the prelimbic cortex (PrL) and orbitofrontal cortex (LO, Figures 3A, 4J). Sparse cell labeling is also observed in the perirhinal cortex (PRh, Figures 3K–P, 4K), ectorhinal cortex (Ect, Figures 3K–P), and temporal association cortex (TeA, Figure 3N).

### 3.3 Brain-wide afferent connections of the BMpc

**Figure 5:**
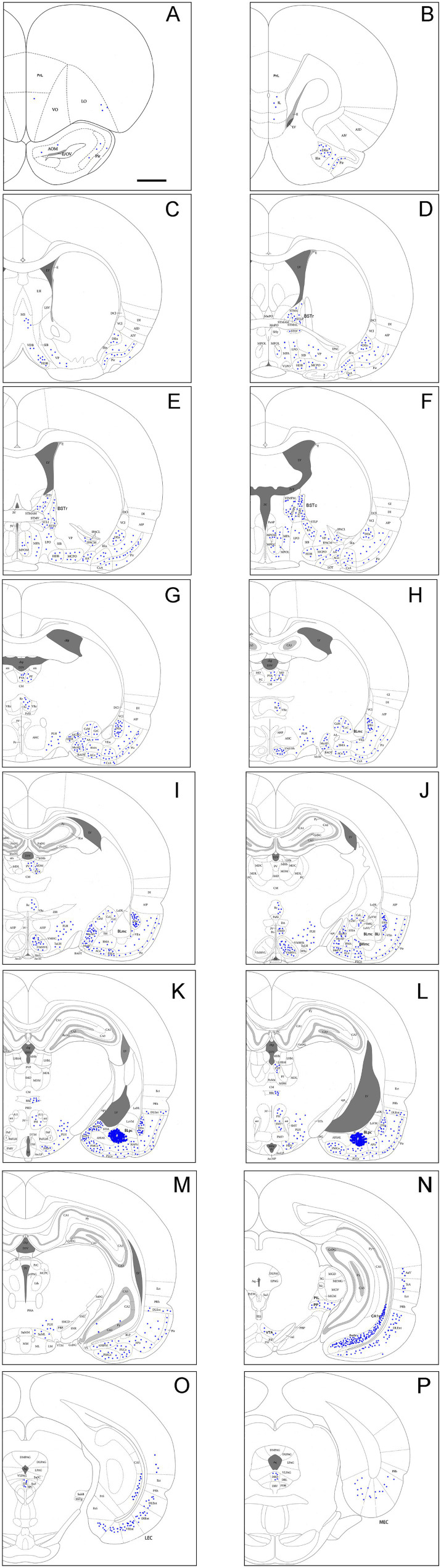
Brain-wide afferent projections of the BMpc in the rat. **(A–P)** Schematic illustration of FG-labeled neurons in sequential anterior **(A)** to posterior **(P)** coronal sections following an FG injection into the BMpc. The injection site (#) is shown as the crowded dot region in panels **(K,L)**. See text for explanation. For abbreviations see the list. Scale bar: 2 mm (applies to all panels).

**Figure 6:**
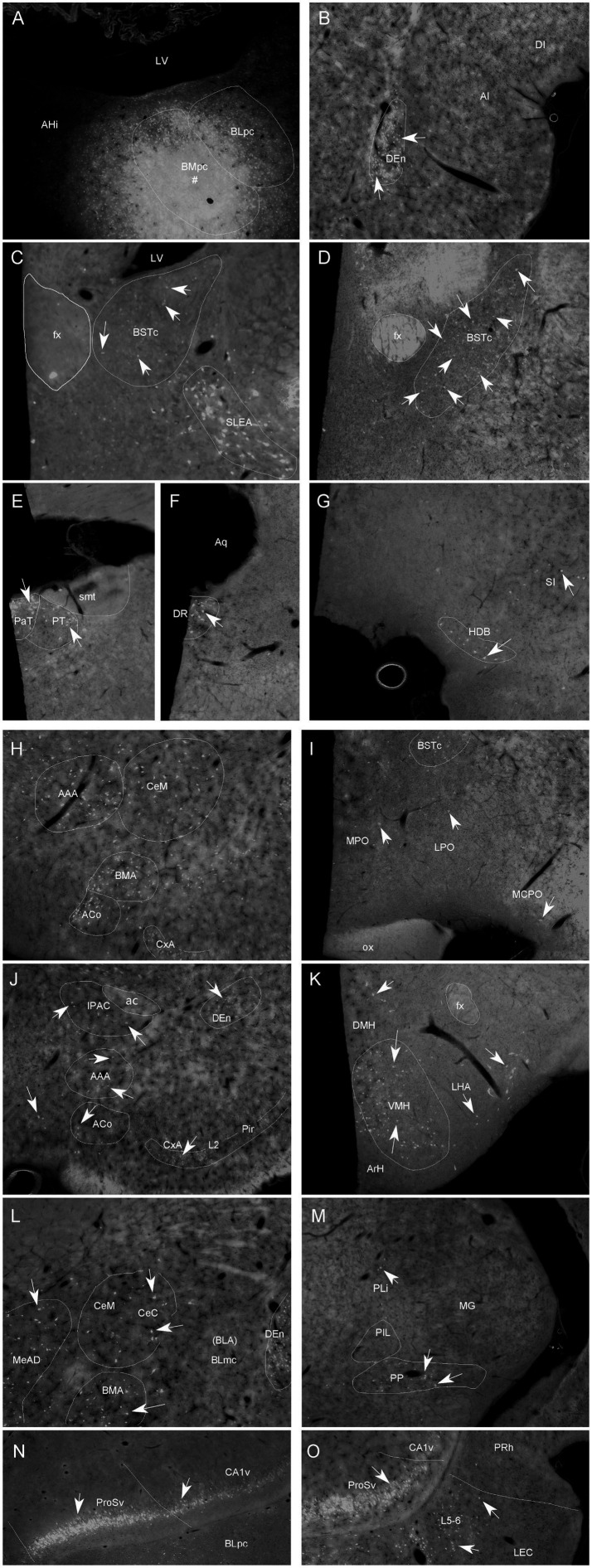
Representative brain regions that send afferent projections to the BMpc in the rat. **(A)** One FG injection site (#) restricted mostly in the BMpc (see the ventral LV). **(B–O)** FG-labeled neurons in the DEn **(B)**, BSTc, SLEA **(C)**, BSTc, **(D)**, PaT, PT **(E)**, DR **(F)**, HDB, SI **(G)**, ACo, BMA, AAA, CeM **(H)**, MPO, LPO **(I)**, IPAC, CxA, ACo, DEn **(J)**, DMH, VMH, LHA **(K)**, CeM, CeC, MeAD, BMA **(L)**, PP **(M)**, ProSv, CA1v, PCo, PAC **(N)**, APir and LEC **(O)**. Arrows indicate some labeled neurons. For abbreviations see the list. Scale bar: 500 μm in **(A)** (applies to all images).

Since the BMpc and BLpc abut with each other, it is relatively difficult to restrict FG injections to the BMpc. In this study, three cases with injections centered in the BMpc are selected for analysis. Figures 5A–P demonstrates brain-wide distribution of FG labeled neurons in one representative case. The injection site is largely located in the BMpc with possible slight involvement in the BLpc (see injection site # in Figures 5K, L, 6A) and FG labeled neurons are mainly found in the ventral part of the brain hemisphere (Figures 5A–P).

Specifically, FG labeled cells are observed in the Pir and CxA (Figures 5A–M, 6J), DEn (Figures 5B–L, 6B), basal forebrain (MS, VDB and HDB, VP/SI) (Figures 5C-F, 6G), IPAC (Figures 5G, 6J), anterior hypothalamic regions (Figures 5D-F, 6I), posterior lateral hypothalamic area (PLH or LHA) (Figures 5G–M, 6K), VMH (Figures 5H–J, 6K), DMH, PH, PMv and SUM (Figures 5J–M).

Many labeled neurons are found in the amygdaloid complex, including the AAA, ACo (Figures 5E-G, 6H,J), all subdivisions of the Me (MeAD, MeAV, MePD and MePV) (Figure 5G-K; Figure 6L), posterior cortical nucleus (PCo/PMCo) and PAC (PLCo) (Figures 5I–M, 6N), as well as in the posteromedial part of the AHi (AHiPM; Figure 5M). There are also labeled neurons in the EA/SLEA (Figures 5F, 6C), BSTr (Figures 5D,E), BSTc (Figures 5F, 6D) and Ce (CeM, CeL and CeC) (Figures 5H–J, 6H,L). Sparsely labeled neurons are also found in the APir (Figures 5K–M) and in a recently defined subdivision of the BST (BSTsc, as defined in Ding, 2023).

In the thalamus, some FG labeled cells are detected in the Re-RRe, PaT (PV), PT (Figures 5G–L, 6E) and PP (Figures 5N, 6M). In the brainstem, retrogradely labeled cells are mainly seen in the DR (Figures 5O,P, 6F).

In the hippocampal regions, the ProSv and adjoining CA1v contain many FG-labeled neurons (Figures 5N, 6N,O). In the entorhinal cortex, many and fewer labeled neurons are found in the deep layers (L5-6) of the LEC and MEC, respectively (Figures 5N–P, 6O).

In the neocortical regions, a few labeled neurons are detected in the orbitofrontal cortex (LO) and infralimbic cortex (IL) (Figures 5A,B).

### 3.4 Comparison of afferent connections of the BLpc and BMpc

Brain-wide semi-quantitative analysis is performed in 6 cases, in which FG injection was mostly restricted to the BLpc (3 cases) or BMpc (3 cases). Overall, this analysis is consistent with the above observation about relatively strength of the afferent connections of the BLpc and BMpc (see Supplementary Table 1). There are both common and differential inputs to the BLpc and BMpc. For example, LEC and ProSv contain many labeled neurons following FG injections into the BLpc or BMpc. In contrast, APir shows many and much fewer labeled neurons after FG injections into the BLpc and BMpc, respectively. Additionally, the PaT and PT contain more labeled neurons following FG injections into the BMpc and BLpc, respectively.

**Figure 7:**
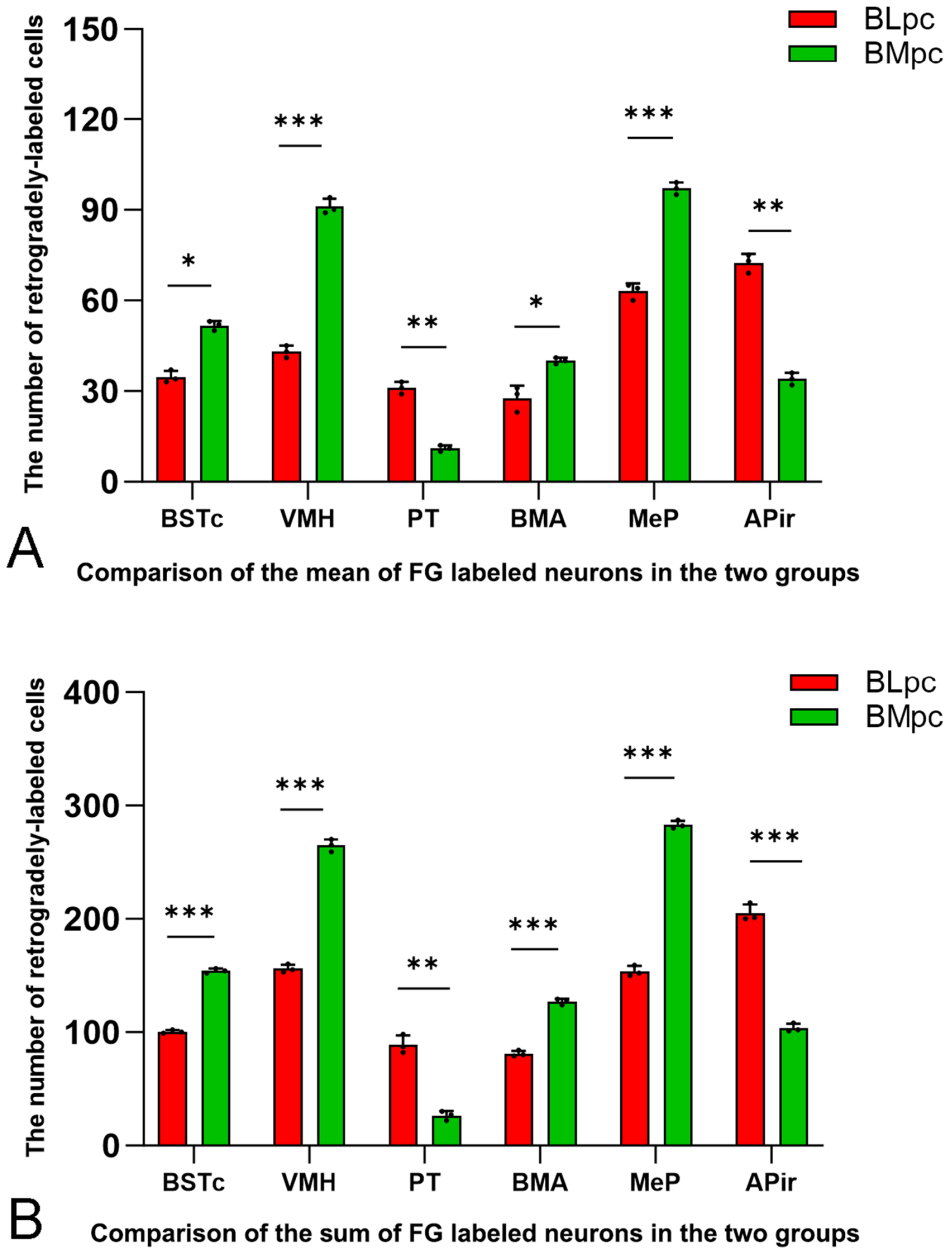
Quantitative comparison of FG-labeled neurons in six input brain regions. Compared are the average number (mean) **(A)** and sum **(B)** of the labeled neurons in the six regions following FG injections into the BLpc and BMpc. Significant difference between the two groups is indicted by *p < 0.05, **p < 0.01 and ***p < 0.001.

Based on the results of semi-quantitative evaluation, FG-labeled neurons in the following brain regions were counted, and these include BSTc, VMH, PT, BMA, MeP and APir. Statistical analysis indicates that the average number (mean) and sum of the labeled neurons in these regions following the BLpc and BMpc injections are significantly different (see Figure 7; *p < 0.05, **p < 0.01, and ***p < 0.01). Specifically, significantly more FG-labeled neurons are observed in the PT and APir following the BLpc injections. In contrast, significantly more labeled neurons are found in the BSTc, VMH, BMA and MeP after the BLpc injections.

**Figure 8:**
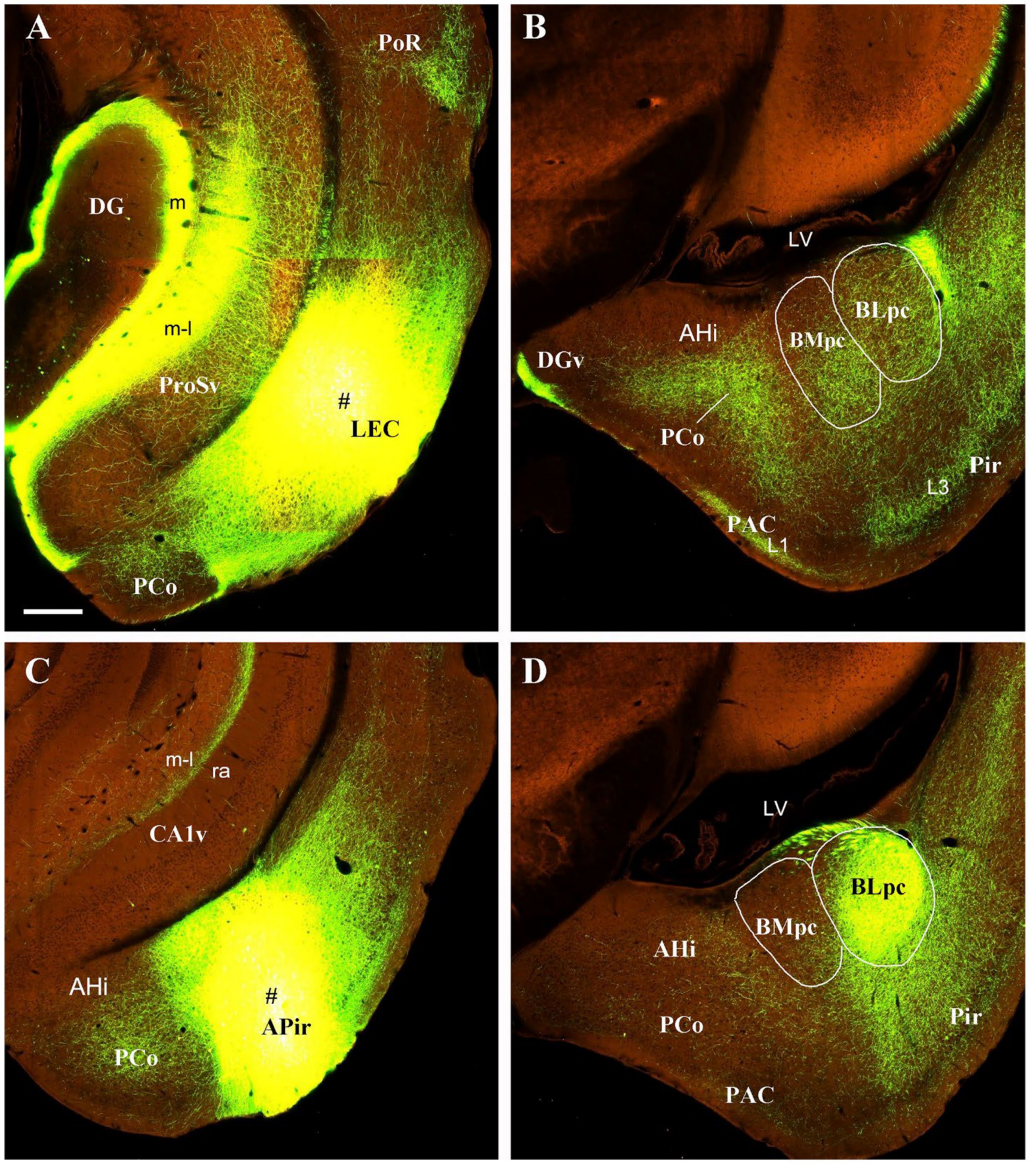
Projections from the LEC and APir to the BLpc and BMpc in the wild-type mice. Raw data are derived from the Allen Institute website (www.brain-map.org) and are revealed with anterograde viral tracing. **(A,B)** Following the tracer injection into the LEC (# in A), resulted axon terminals are observed in both BLpc and BMpc **(B)**. **(C,D)** Following the tracer injection into the APir, resulted axon terminals are seen mostly in the BLpc. Scale bar: 280 μm in **(A)** (applies to all panels).

**Figure 9:**
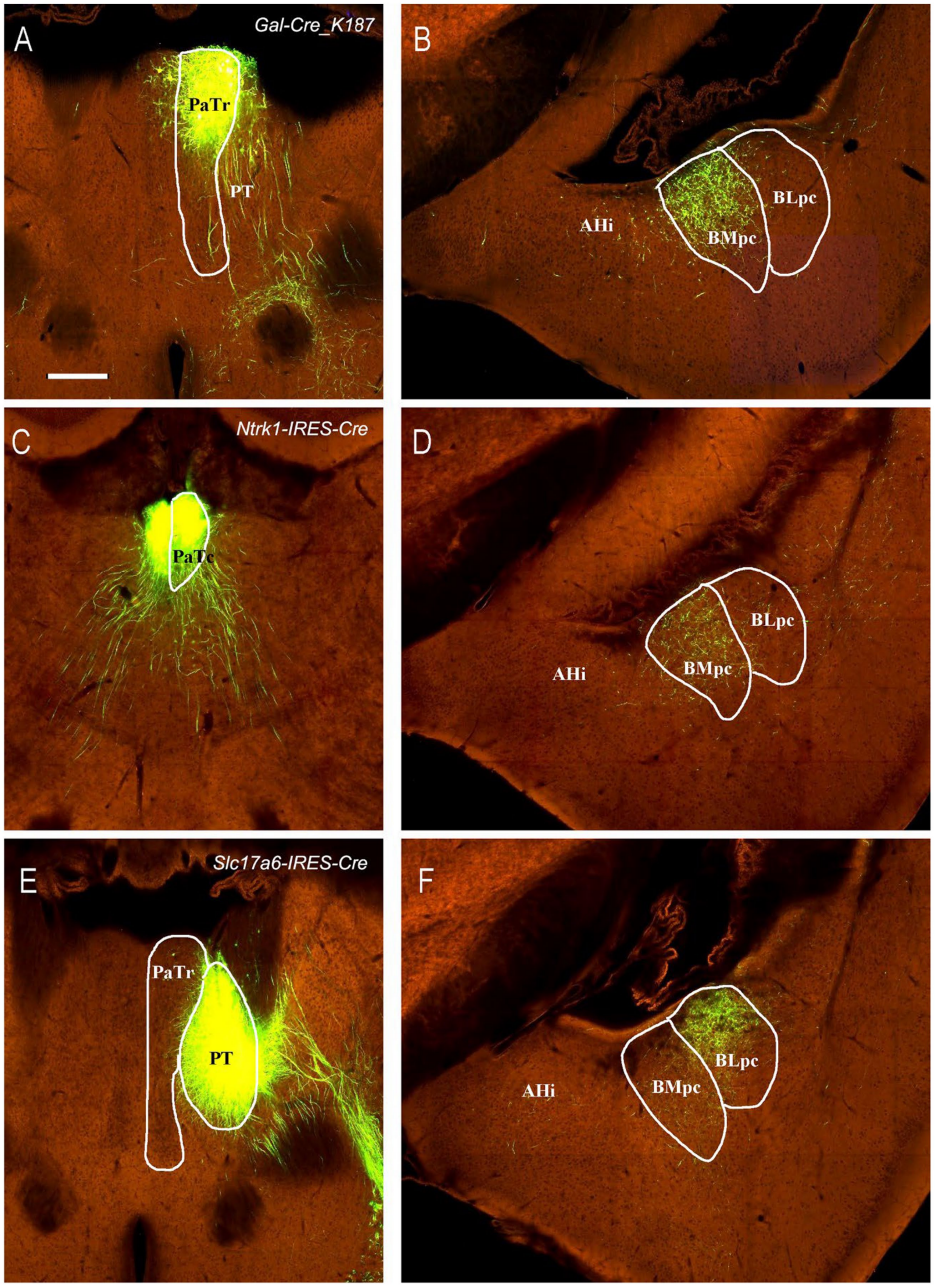
Projections from the PaT and PT to the BLpc and BMpc in the Cre-line mice. Raw data are derived from the Allen Institute website (www.brain-map.org). **(A–D)** Following the tracer injections into the PaTr (# in A) and PaTc (# in C), resulted axon terminals are found mostly in the BMpc **(B,D)**. **(E,F)** Following the tracer injection into the PT (# in **E**), resulted axon terminals exist mostly in the BLpc **(F)**. Scale bar: 280 μm in **(A)** (applies to all panels).

**Figure 10:**
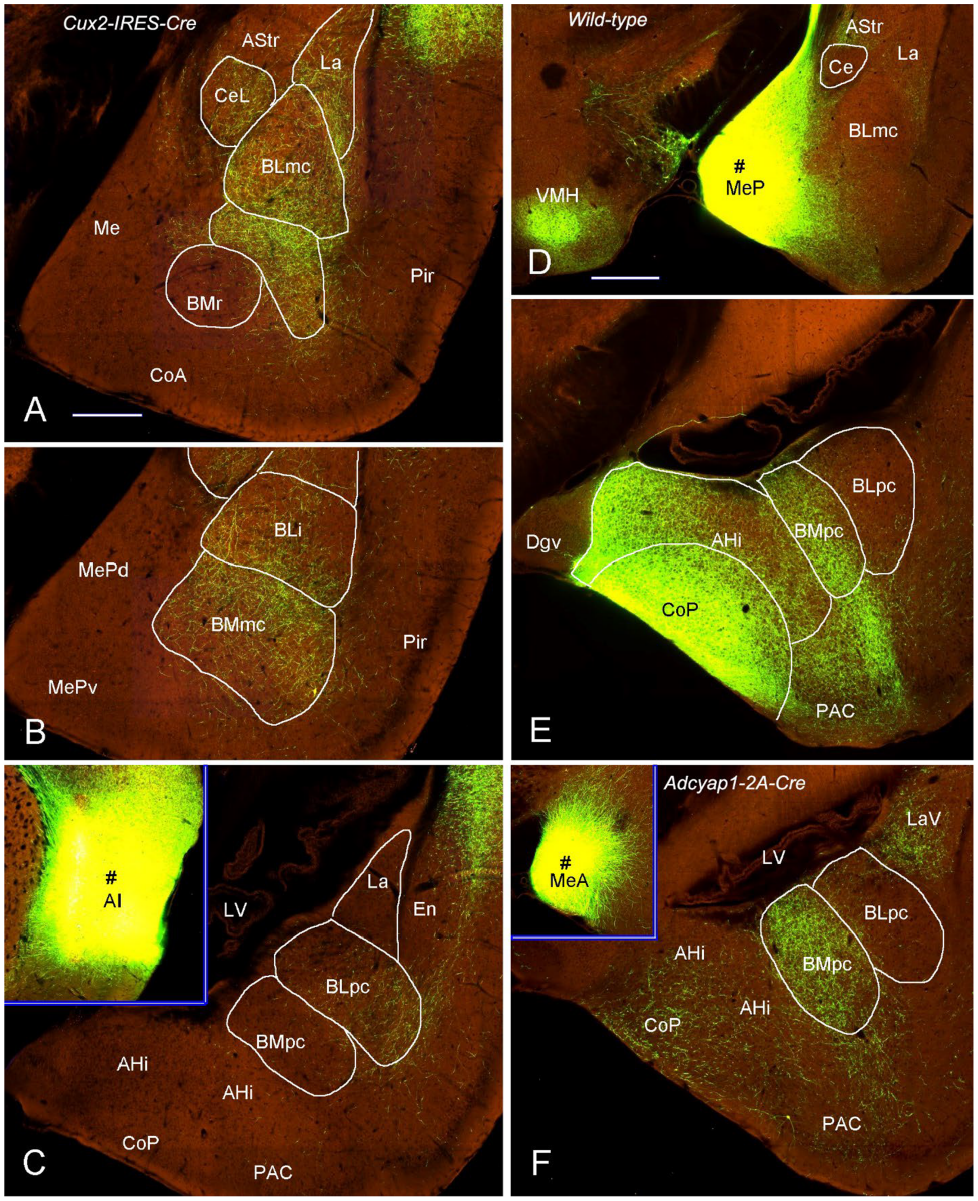
Projections from the AI and Me to the BLpc and BMpc in the Cre-line mice. Raw data are derived from the Allen Institute website (www.brain-map.org). **(A–C)** Differentially labeled axon terminals in the BL and BM subdivisions after the tracer injections into the AI (# in the inset of panel C). All the BL subdivisions (BLmc, BLi and BLpc) contain labeled terminals but only the BMmc **(A,B)** has labeled terminals. Both BMr **(A)** and BMpc **(C)** contain almost no terminal labeling. **(D,E)** Following the tracer injection into the MeP (# in **D**), resulted axon terminals are mainly seen in the BMpc with only faint labeling in the BLpc **(E)**. It is noted that strong terminal labeling is also observed in the VMH **(D)**, CoP and AHi **(E)**. **(F)** Labeled axon terminals are mainly found in the BMpc but not the BLpc after the tracer injection into the MeA (# in the inset of panel **F**). Scale bars: 350 μm in **(A)** (applies to panels **A–C,E,F**); 560 μm in **(D)** (applies to panel **D** and the insets in **C,F**).

To further confirm these retrograde tracing results with an anterograde tracing method, we have analyzed some mouse connectional data derived from the Allen Institute website (www.brain-map.org). As expected, an anterograde viral tracer injection into the LEC results in many labeled axon terminals in both BLpc and BMpc (Figures 8A,B). In contrast, the injection into the APir leads to heavy terminal labeling mainly in the BLpc with much fewer labeled terminals in the BMpc (Figures 8C,D). The tracer injections into the anterior and posterior PaT (PaTr and PaTc) produce labeled terminals mainly in the BMpc with fewer labeling in the BLpc (Figures 9A–D). In contrast, the injection into the PT results in terminal labeling mainly in the BLpc with fewer in the BMpc (Figures 9E,F). Furthermore, the tracer injection into the AI produces labeled axon terminals in the BLmc, BLi and BLpc as well as in the BMmc but not in the BMr and BMpc (Figures 10A–C). Additionally, the injections into the MeP and MeA lead to densely labeled terminals in the BMpc with much fewer labeling in the BMpc (Figures 10D–F).

### 3.5 Brain-wide efferent projections of the BLpc and BMpc

**Figure 11:**
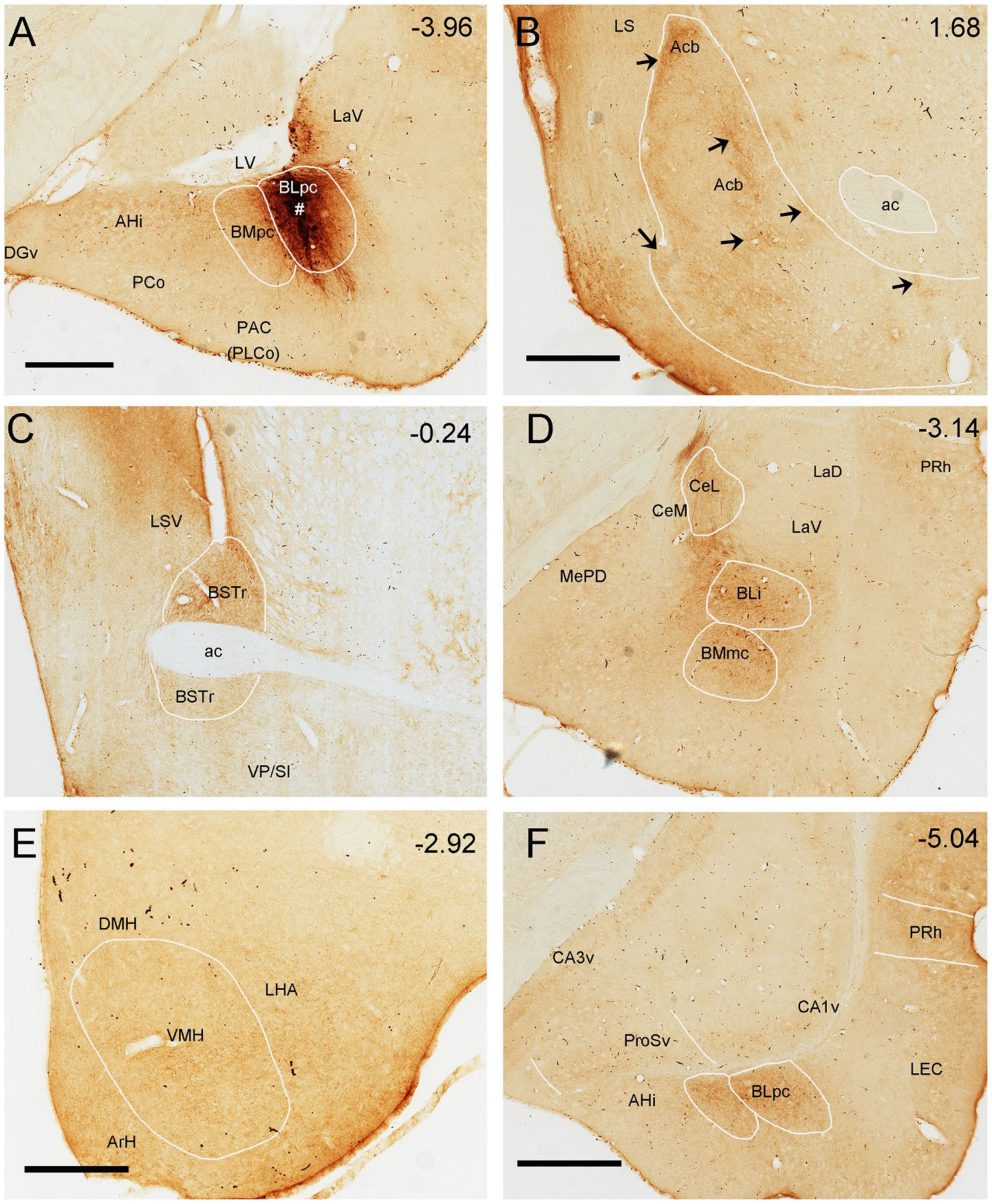
Efferent projections of the rat BLpc. **(A)** One BDA injection site (#) restricted in the BLpc (near the LV). **(B-F)** BDA-labeled axon terminals in the dorsomedial Acb (**B**; arrows indicate terminal clusters), BSTr **(C)**, CeL, BLi and BMmc **(D)**, VMH, LHA **(E)**, PRh and posterior BLpc **(F)**. Approximate bregma coordinates are indicated in the upper right corner of each panel. For abbreviations see the list. Scale bars: 700 μm in **(A)** (applies to **A,C,D**); 600 μm in **(B)**; 400 μm in **(E)**; 800 μm in **(F)**.

The anterograde tracer BDA is successfully injected into the BLpc in three rats to explore the efferent connections of the BLpc. As an example, shown in Figure 11, one BDA injection into the BLpc leads to moderately labeled axon terminals mainly in the AHi, BMpc (Figure 11A), nucleus accumbens (Acb) (see the patch-like terminal fields in Figure 11B), BSTr (Figure 11C), BMA (BMr), BLi (Figure 11D) and CeL (Figure 11D). Weak terminal labeling is also seen in the VMH and LHA (Figure 11E), PRh-Ect (Figure 11F), ProSv-CA1v (Figure 11F), LEC, PrL-IL, AI-DI and CeM (Supplementary Table 1).

**Figure 12:**
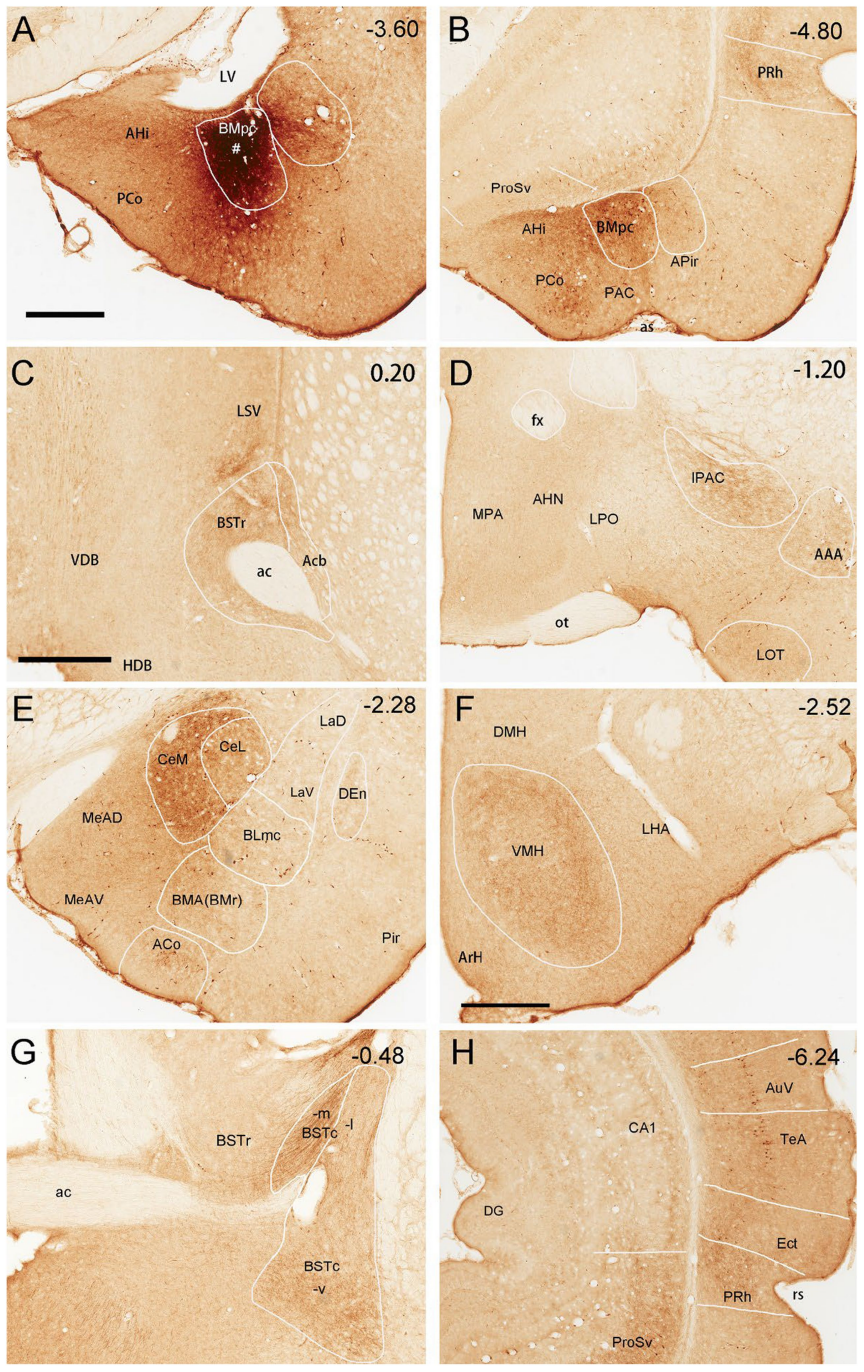
Efferent projections of the rat BMpc. **(A)** One BDA injection site (#) restricted in the BMpc (near the LV). **(B–H)** BDA-labeled axon terminals in the AHi, PCo, BMpc, anterior PRh **(B)**, anterior BSTr, LSV **(C)**, IPAC, AAA **(D)**, CeM, CeL, BMA **(E)**, VMH, LHA **(F)**, BSTc **(G)**, ProSv and posterior PRh (H). Note that some retrogradely labeled neurons are also observed in the ProSv **(B)**, ventral auditory association cortex (AuV), and temporal association cortex (TeA) **(H)**. Approximate bregma coordinates are indicated in the upper right corner of each panel. For abbreviations see the list. Scale bars: 700 μm in **(A)** (applies to **A,B,D,E,G,H**); 600 μm in **(C)**; 500 μm in **(F)**.

To reveal brain-wide efferent connections of the BMpc, the tracer BDA is successfully injected into the BMpc in four rats, in which the tracer is confined to the BMpc. Figure 12 demonstrates major target regions of the BMpc projections in one representative case. In this and other cases, the BDA injection is mostly restricted in the BMpc (e.g., Figure 12A; Supplementary Figure 1) and produces dense axon terminal labeling in the AHi, BMpc, PCo, PRh-Ect (Figure 12B), LSV, the most rostral BSTr (Figure 12C), IPAC, AAA (Figure 12D), CeM (Figure 12E), VMH (Figure 12F), BSTc (Figure 12G), ProSv (Figure 12H). Very weak terminal labeling is also detected in the LHA, olfactory tubercle (Tu), BSTc, MePD, APir, LOT, BMA, La, PrL and IL (Supplementary Table 1; Supplementary Figure 3). Additionally, in this case, some BDA-labeled neurons are also noted in the ProSv (Figure 12B) and the temporal cortical areas (AuV and TeA; Figure 12H).

**Figure 13:**
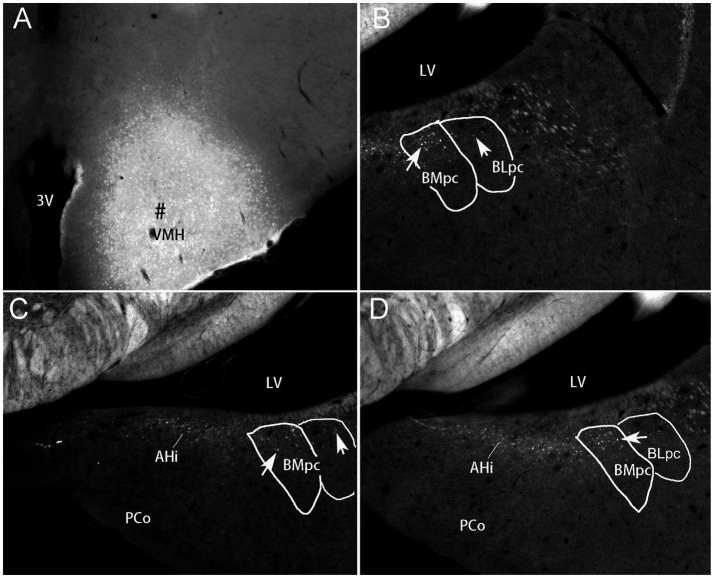
BMpc projections to the VMH revealed with FG retrograde tracing. **(A)** One FG injection site (#) in the VMH of the rat. **(B–D)** Anterior **(B)** to posterior **(D)** sections showing FG-labeled neurons in the BMpc and AHi with much fewer in the BLpc **(B)**. Arrows indicate some labeled neurons. Scale bar: 500 μm in **(A)** for all panels.

To confirm the efferent projections from the BMpc to VMH with a retrograde tracing method, we have injected FG into the VMH in three rats (e.g., Figure 13A). The results show that many FG-labeled neurons are observed in the BMpc with fewer neurons in the BLpc (Figures 13B–D). Additionally, many labeled neurons are also seen in the AHi (Figures 13B–D).

To compare the efferent projections of the BLpc and BMpc, brain-wide semi-quantitative rating of BDA-labeled axon terminals in their target regions is performed in 6 cases (3 cases for each group). Overall, both regions strongly innervate Acb, BSTr, AHi, CeL, Ect and PRh. The BLpc preferentially targets BMmc and BLi while the BMpc sends its efferent connections preferentially to LSV, BSTc, IPAC, AAA, ACo, PCo, CeM, VMH, PAC and ProSv (see Supplementary Table 1).

## 4 Discussion

Some previous connectional studies on rat BL (or B) and BM (or AB) treat the BL or BM as single entity (e.g., Veening, 1978a; Veening, 1978b; Ottersen, 1980; Kelley et al., 1982; Mcdonald, 1987). More previous studies differentiate the anterior (BLA; large cells) and posterior (BLP; smaller cells) parts of the BL and/or the anterior (BMA; very small cells) and posterior (BMP; larger cells) portions of the BM (e.g., Krettek and Price, 1977a; Mcdonald, 1984; De Olmos et al., 1985; Price et al., 1987; Savander et al., 1996; Petrovich et al., 1996; Paxinos and Watson, 2007). In the studies on the BLP or BMP, the tracer injections are mainly placed in the anterior BLP (i.e., BLi) or anterior BMP (i.e., BMmc) with no or little involvement in the BLpc or BMpc defined in the present study. The terms BLpc and BMpc are modified from Savander et al. (1995) (for BL) and Pikkarainen and Pitkänen (2001) (for BM). Overall, the BLpc and BMpc are the most posterior parts of the BLP and BMP, respectively (see Figures 1, 2 and Supplementary Figure 2). In practice, the BLpc and BMpc start anteriorly at the level where the lateral ventricle (LV) appears ventrally and closely adjoins the posterior amygdala in coronal sections (e.g., Figure 1B). Since connectional data about these BLpc and BMpc are very limited, the present study focuses on brain-wide connections of these two regions in rats. We find that the BLpc and BMpc have differential connections and are also different from other subdivisions of the BL and BM. More importantly, the BLpc and BMpc display many comparable connections to monkey BLpc and BMpc, respectively (see discussion below).

### 4.1 Common and differential connections of the rat BLpc versus BLi or BLmc

**Figure 14:**
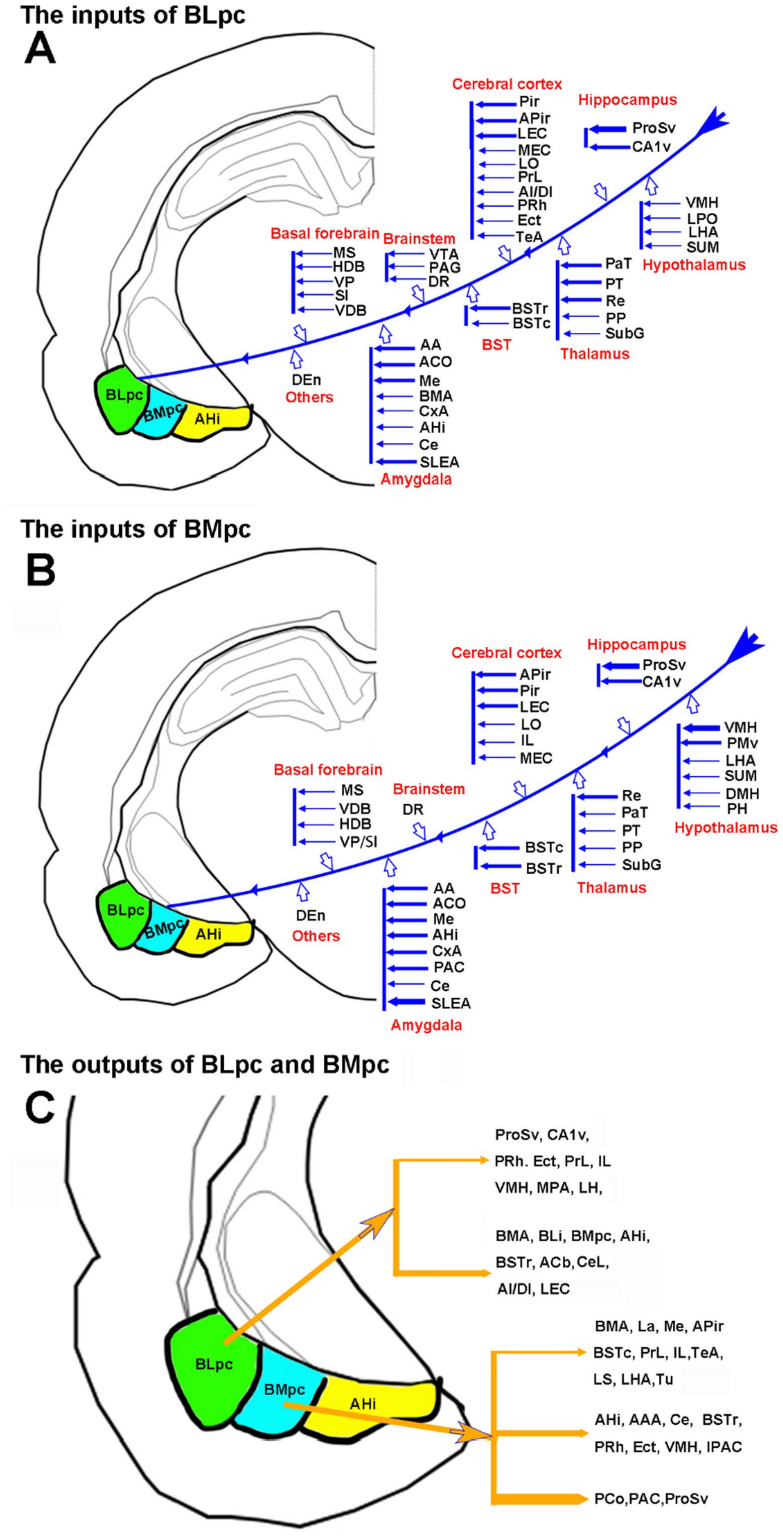
Summary of the connectivity of the BLpc and BMpc. **(A)** Afferent projections of the BLpc. **(B)** Afferent projections of the BMpc. **(C)** Efferent projections of the BLpc and BMpc. The thicker and thinner arrows indicate stronger and weaker projections, respectively.

The present study has shown that strong inputs of the BLpc come mainly from the ProSv, CA1v, APir, DEn with weaker to moderate projections from the Pir, BST, PaT, PT, Re and PP (see Figure 14A for summary). In contrast, the BLi-BLmc receives strong inputs from many limbic cortices such as the PrL-IL, AI, DI, PRh-Ect and LEC (Wyss, 1981; Ottersen, 1982; Sesack et al., 1989; Hurley et al., 1991; Romanski and LeDoux, 1993; Mcdonald, 1998; Shi and Cassell, 1998, 1999) as well as from the hippocampal regions, CA1 and “subiculum” (Ottersen, 1982; van Groen and Wyss, 1990; Canteras and Swanson, 1992; Mcdonald, 1998; Shi and Cassell, 1999). The so-called “subiculum” in previous rodent literature probably corresponds to the ProS based on comprehensive analysis of multimodal data (for details, see Ding, 2013; Ding et al., 2020). The subcortical regions projecting to the BLi-BLmc contain the PaT-PT (Veening, 1978a, 1978b; Nitecka et al., 1979; Ottersen and Ben-Ari, 1979; Woolf and Butcher, 1982; Turner and Herkenham, 1991), MD (van Vulpen and Verwer, 1989); Cla (Majak et al., 2002), DR (Vertes, 1991) and APir (Santiago and Shammah-Lagnado, 2005). Therefore, common and major differences in afferent connections exist between the BLpc and BLi-BLmc.

As for the output projections of the rat BLpc, the present study has revealed very limited regions that receive strong inputs from the BLpc. These regions mainly include the mediodorsal Acb, BSTr, BLi, CeL, BMmc and AHi (see Figure 14C for summary) with no or few efferent terminals in the PaS and LOT. In sharp contrast, BLi-BLmc sends widespread and strong projections to many cortical and subcortical regions such as PrL-IL, AI, LEC, ProS-CA1, AHi, PaS, CPu, Acb-OT, Cla, BST, SI, and LOT (Krettek and Price, 1977a, 1977b, 1978a, 1978b; Ottersen, 1982; Kelley et al., 1982; Weller and Smith, 1982; Russchen and Price, 1984; Sripanidkulchai et al., 1984; Luiten et al., 1987; McDonald and Jackson, 1987; Kita and Kitai, 1990; van Groen and Wyss, 1990; Mcdonald, 1991a, 1991b; Savander et al., 1995; Wright and Groenewegen, 1995, 1996; Pikkarainen et al., 1999; Ding, 2013). Therefore, the major difference in efferent projections between the BLpc and BLi-BLmc is two folds. First, the latter sends much heavier projections to many of its target regions than the former does. Second, the latter rather than the former projects very strongly to the PaS and LOT, which could be used to evaluate if anterograde tracer injections are involved in the BLi-BLmc or BLpc or both in future studies.

### 4.2 Common and differential connections of the rat BMpc versus BMmc or BMr

In this study, we have revealed that major afferent connections of the BMpc originate from the ProSv-CA1v, LEC, VMH, BST, Me and DEn with weaker projections from many other regions including the Pir, MPN, APir, LHA, PaT-PT, La and DR (see Figure 14B for summary). As a comparison, projections to the BMmc-BMA region arise from the ProS-CA1, PRh-Ect, LEC, IL, AI, Pir, PaT and La (Ottersen and Ben-Ari, 1979; Ottersen, 1982; Hurley et al., 1991; Takagishi and Chiba, 1991; Canteras and Swanson, 1992; Romanski and LeDoux, 1993; Moga et al., 1995; Mcdonald et al., 1996; Mcdonald and Mascagni, 1997; Mcdonald, 1998; Savander et al., 1997; Shi and Cassell, 1998, 1999). In summary, both common and differential inputs are found to the BMpc and/or BMmc-BMA. It is also noted that, compared to the BMmc, the BMpc receives much stronger and weaker projections from the Me and ProSv-CA1v, respectively.

For the efferent projections of the rat BMpc, the present study shows that the BMpc gives rise to moderate-strong projections to the AHi, PCo, BSTr, IPAC, CeM, VMH, PRh-Ect and ProSv (see Figure 14C for summary). This finding is consistent with a previous study, in which an injection placed in the posterior BMP (corresponding to the BMpc in the present study) results in axon terminal labeling mainly in the regions mentioned above (Petrovich et al., 1996). Compared to the BMpc, the region corresponding to the BMmc projects heavily to the ProSv-CA1v, IL, PRh-Ect, VMH, BST, SI, Acb, and Pir (Krettek and Price, 1978a, 1978b; Kelley et al., 1982; Russchen and Price, 1984; Ono et al., 1985; Mcdonald, 1987; Mcdonald, 1991a, 1991b; Savander et al., 1996; Wright et al., 1996; Pikkarainen et al., 1999; Majak et al., 2004). It is also reported that the BMA projects mainly to the BST, CeM, and APir with no projections to the ProSv-CA1v (Petrovich et al., 1996). Therefore, the BMmc, BMpc and BMA send very strong, strong and faint projections to the ProSv-CA1v, respectively. Additionally, the AI in mice projects heavily to the BMmc with almost no labeling to the BMpc (see Figures 10A–C). These are all consistent with the concept of at least three major subdivisions of the BM in rodents defined in the present study.

### 4.3 Comparable connectivity of the BLpc in the rats and monkeys

As mentioned in “Introduction” section, although human and monkey BLpc is localized in the ventral part of the BL, rodent BLpc exists in the caudal part of the BL because this BLpc is the only region that contains much smaller cells than the rostral (large cells) and intermediate (medium-sized cells) parts of the BL. In the present study, the BLpc mainly projects to the Acb, BSTr, CeL, BLi, BMmc, BMpc, and AHi with weak projections to PrL-IL, LO, AI-DI and some hypothalamic regions (e.g., VMH and LHA). Similarly, the BLpc in the monkeys send efferent projections mainly to ventromedial prefrontal cortex (areas 24 and 25; Porrino et al., 1981; Sharma et al., 2020), anterior insular cortex (AI and DI; Cho et al., 2013), orbitofrontal cortex (e.g., areas 14 and 12; Barbas and De Olmos, 1990; Carmichael and Price, 1995), entorhinal cortex (LEC; Pitkänen et al., 2002), perirhinal-ectorhinal cortex (areas 35 and 36 or PRh and Ect; Saunders and Rosene, 1988; Stefanacci et al., 1996) and ProS-CA1 (Aggleton, 1986; Saunders et al., 1988; Fudge et al., 2012). The BLpc in monkeys also innervates subcortical regions such as Acb and other ventral striatal regions (Friedman et al., 2002; Fudge et al., 2002; Decampo and Fudge, 2013; Choi et al., 2017), BSTr and SLEA (Decampo and Fudge, 2013), CeM-CeL (Fudge and Tucker, 2009), and MD (Russchen et al., 1987). In summary, the rodent equivalent of the primate BLpc proposed in this study is further supported by similar efferent connections of the BLpc in the rodents and monkeys.

The BLpc in the rats and monkeys also appear to have similar afferent projections. The present study has revealed that the BLpc in the rats receives its main cortical inputs from the ProS-CA1, PrL-IL, Pir, AI-DI, PRh-Ect and LEC, and its subcortical inputs from the MS-NDB-SI, La, BST, DEn, PaT-PT, PP, VMH, LHA, DR and PAG. In general, these results are consistent with those reported in monkeys. Specifically, the monkey BLpc is mainly innervated by the inputs from the prepiriform cortex (area 51-part of piriform cortex; Van Hoesen, 1981), ProS-CA1 (Rosene and van Hoesen, 1977; Van Hoesen, 1981; Aggleton, 1986; Saunders et al., 1988; Fudge et al., 2012), ventromedial prefrontal cortex (areas 32, 24 and 25, roughly equivalent to the PrL and IL in rodents) (Freedman et al., 2000; Chiba et al., 2001; Pikkarainen and Pitkänen, 2001; Stefanacci and Amaral, 2002; Cho et al., 2013), agranular and dysgranular insular cortex (i.e., AI-DI) (Turner et al., 1980; Stefanacci and Amaral, 2002; Cho et al., 2013) and areas 35 and 36 (i.e., PRh-Ect) (Herzog and van Hoesen, 1976; Stefanacci et al., 1996). Subcortical inputs from hypothalamus (e.g., VMH and LHA; Amaral et al., 1982), thalamus (e.g., PaT-PT and PP), basal forebrain (SI, VDB and HDB), brainstem (e.g., PAG) (see Aggleton et al., 1980) and other amygdaloid regions (e.g., La and BLi) (Pitkänen and Amaral, 1998; Bonda, 2000) are also reported in monkeys. These similar inputs to the BLpc in rodents and monkeys further support our conclusion that rodent BLpc is the posterior part of the BLP, which was not further subdivided in rodent literature.

### 4.4 Comparable connectivity of the BMpc in rats and monkeys

The BMpc lies medioventral to the BLpc. As shown in this study, the main sources of the BMpc inputs are derived from the ProSv-CA1v, Pir, IL, LEC, APir, La, BST-SLEA, PaT-PT, VMH, LHA, MeA, DEn, and DR. Weaker afferents also originate from the AI -DI, PrL, Re, basal forebrain (MS, LS, HDB, VDB, SI), PP-MGM, AON, Acb-OT, Cla, PAG, VTA, and other amygdaloid regions (CoP, BMA, BMmc, BLA and Ce). As for monkeys, there is only limited literature available about the inputs of the BMpc. For example, the monkey BMpc receives its connections from area 25 and parainsula (Freedman et al., 2000; Stefanacci and Amaral, 2002), from areas 35 and 36 (i.e., PRh-Ect) (Savander et al., 1996), La (Pitkänen and Amaral, 1998), Me (Amaral et al., 1992) and hypothalamus (VMH, LHA) (Amaral et al., 1992). Overall, these inputs to the monkey BMpc are comparable to those to the rat BMpc.

Rat BMpc originates its main output projections to the AHi, PCo, BSTr, IPAC, CeM, VMH, PRh-Ect and ProSv. Consistently, the monkey BMpc sends its projections to cortical areas 35 and 36 (Stefanacci et al., 1996), insular areas (AI-DI), orbitofrontal cortex (Barbas and De Olmos, 1990; Carmichael and Price, 1995), and the hippocampal region (ProS-CA1; Aggleton, 1986; Saunders et al., 1988; Bonda, 2000; Fudge et al., 2012; Ding, 2013), as well as to some subcortical regions such as Acb (Friedman et al., 2002; Decampo and Fudge, 2013), Ce, IPAC (Fudge and Tucker, 2009), and BSTr (Decampo and Fudge, 2013).

### 4.5 Functional consideration of the BLpc and BMpc

The basolateral nuclear complex consists of the BL, BM, and La. The present study focuses on the most posterior part of the BL and BM. The BL is widely connected with limbic cortical regions such as the medial prefrontal cortex, perirhinal and insular areas and hippocampal formation, as well as the limbic subcortical regions such as Acb-OT, Ce, and midline thalamic regions. The connections between the BL and Acb-OT are associated with rewards while the BL connections with the BST and Ce are closely related to the generation of anxiety and fear. In addition, the connections of the BL with the hippocampus (mainly ProSv-CA1v) are associated with emotion-related memory (Yang and Wang, 2017; Mcdonald, 2020). It is interesting to note that recent studies have found that the neural network of the BLA and BLP is completely different. The BLA (BLmc) innervates the deep part of the ProSv-CA1v pyramidal layer while the BLP (BLi and BLpc) innervates the superficial part of the ProSv-CA1v pyramidal layer. Moreover, the BLP-CA1v inputs antagonize anxiety while the BLA-CA1v inputs cause anxiety (Pi et al., 2020). In addition, excitatory inputs and activation of BLP-CA1v lead to epileptic seizures, and the BLP is a key control point for controlling temporal lobe epilepsy (Sun et al., 2024). The findings in the present study suggest that the BLpc has much fewer connections with its target regions compared to the BLmc and BLi. However, the BLpc does send relatively strong connections to the Acb, BSTr and BMmc, indicating that it is involved in reward, anxiety and memory functions.

Compared to the BL, there are fewer functional studies on BM-related circuits. Recent studies have found that the BM and BST can transmit information to the VMH and target different domains within the VMH, thereby regulating innate defensive behavior (Yamamoto et al., 2018). This is consistent with our finding of strong BMpc projections to the VMH. It has also been reported that simultaneous or separate inactivation of the BM and BL can reduce the expression of fear, and this regulation has a synergistic effect (Amano et al., 2011). Another study has found that the projections from the BM to the medial intercalated cells in mice can reduce fear memory (Rajbhandari et al., 2021). Strong projections from the BMpc to the BST, IPAC, CeM and ProSv-CA1v revealed in the present study suggest that the BMpc is also involved in the generation of anxiety, fear and memory. Structural and connectional differences of the BM along the anterior–posterior axis can form different neural circuits, thereby mediating different aspects of emotions and socially related behaviors (Petrovich et al., 1996; Hintiryan et al., 2021).

Overall, previous studies on the structure and functions of the BL and BM mostly focus on their anterior and middle parts. The present study has provided deeper insight into the brain-wide afferent and efferent projections of their most posterior part (i.e., BLpc and BLpc). Our findings on the posterior part, together with previous findings on the anterior and middle parts, have displayed the connectional differences between the BL and BM along the anterior–posterior axis in rodents. These findings would provide an important anatomical basis for the understanding of emotion-related neuronal circuits and diseases.

## Data Availability

The original contributions presented in the study are included in the article/Supplementary material, further inquiries can be directed to the corresponding author.

## Supplementary material

SUPPLEMENTARY FIGURE 1 Representative injection sites of the tracers FG and BDA. **(A–F)** Six FG injection sites in the BLpc **(A–C)** and BMpc **(D–F)**. **(G–L)** Six BDA injection sites in the BLpc **(G–I)** and BMpc **(J-L)**. The injection cores are indicated by #. Scale bars: 500 μm in **(A)** for **(A–F)**; 600 μm in **(G)** for **(G–I)**; 800 μm in **(J)** for **(J–L)**.

SUPPLEMENTARY FIGURE 2 Location, cytoarchitecture and subdivisions of the BL and BM in the sagittal sections of mouse brain. **(A,B)** Two adjacent sections stained for Nissl substance **(A)** and Chrm2 expression **(B)** showing the overall cell size **(A)** and Chrm2 expression **(B)** in the BL and BM subdivisions. The anterior and posterior subdivisions in orange color indicate those in most brain atlases while those in black color indicate the terms used in the present study. Note that BLmc and BLi display stronger Chrm2 expression signals than BLpc does. In contrast, BMr (BMA) and BMpc show faint Chrm2 expression whereas BMmc has stronger expression (indicated by arrows). **(C)** One closely matched sagittal section showing the strong Zdhhc7 expression in the BLmc, BLi and BLpc (with a gradient). In contrast, all BM subdivisions display faint expressions. This expression pattern makes the BL and BM stand out. Scale bar: 300 μm in **(A)** (for panels **A–C**).

SUPPLEMENTARY FIGURE 3 Brain-wide efferent projections of the BMpc in the rat. **(A–L)** Schematic illustration of BDA-labeled axon terminals in sequential anterior **(A)** to posterior **(L)** coronal sections following a BDA injection into the BMpc. The injection site (#) is shown as the crowded dot regions (#) in panels **(H,I)**. See text for explanation. For abbreviations see the list. Scale bar: 2 mm (applies to all panels).

SUPPLEMENTARY TABLE 1 Semi-quantitative rating of FG-labeled neurons (inputs) and BDA-labeled axon terminals (outputs) in the rats following the tracer injections into the BLpc (*n* = 3) and BMpc (*n* = 3). Neuronal and axon terminal density rating (-), (+), (++) and (+++) indicate none, low, moderate and high densities, respectively.

## Glossary

3 V: Third ventricle
AAA: Anterior amygdaloid area
ac: Anterior commissure
Acb: Nucleus accumbens
ACo: Anterior cortical amygdaloid nucleus (=CoA)
AHC: Anterior hypothalamic nucleus, central part
AHi: Amygdalohippocampal area
AI: Agranular insular cortex
AIV: Agranular insular cortex, ventral part
AID: Agranular insular cortex, dorsal part
AIP: Agranular insular cortex, posterior part
APir: Amygdalopiriform transition area
ArH: arcuate nucleus of hypothalamus
as: Amygdaloid sulcus
AuV: Secondary auditory cortex, ventral part
BLA: Basolateral amygdaloid nucleus, anterior part
BLP: Basolateral amygdaloid nucleus, posterior part
BMA: Basmedial amygdaloid nucleus, anterior part
BMP: Basomedial amygdaloid nucleus, posterior part
BST: Bed nucleus of the stria terminalis
BSTr: Bed nucleus of the stria terminalis, rostral part
BSTc: Bed nucleus of the stria terminalis, caudal part
CA1v: Field CA1 of the hippocampus, ventral part
CA3v: Field CA3 of the hippocampus, ventral part
Ce: Central amygdaloid nucleus
CeM: Central amygdaloid nucleus, medial division
CeL: Central amygdaloid nucleus, lateral division
CeC: Central amygdaloid nucleus, capsular part
CPu: Caudate putamen (~striatum)
CxA: Cortex-amygdala transition zone
DI: Dysgranular insular cortex
DEn: Dorsal endopiriform nucleus
DIEnt: Dorsal intermediate entorhinal cortex
DLEnt: Dorsolateral entorhinal cortex
DMH: Dorsomedial hypothalamic nucleus
DR: Dorsal raphe nucleus
EA: Extended amygdala.
Ect: Ectorhinal cortex
fx: Fornix
GI: Granular insular cortex
HDB: Nucleus of diagonal band, horizontal limb
ic: Internal capsule.
IL: Infralimbic cortex
IPAC: Interstitial nucleus of the posterior limb of anterior commissure
La: lateral amygdaloid nucleus
LaD: Lateral amygdaloid nucleus, dorsal part
LaV: Lateral amygdaloid nucleus, ventral part
LEC: Lateral entorhinal cortex
LHA: Lateral hypothalamic area
LOT: Nucleus of the lateral olfactory tract
LPO: Lateral preoptic area
LSV: Lateral septal nucleus, ventral part
LV: Lateral ventricle
MCPO: Magnocellular preoptic nucleus
Me: Medial amygdaloid nucleus
MeAD: Medial amygdaloid nucleus, anterodorsal part
MeAV: Medial amygdaloid nucleus, anteroventral part
MePD: Medial amygdaloid nucleus, posterodorsal part
MePV: Medial amygdaloid nucleus, posteroventral part
MG: Medial geniculate nucleus
MPA: Medial preoptic area
MPO: Medial preoptic nucleus
MS: Medial septal nucleus
PAC: Periamygdaloid cortex (~PLCo).
PAG: Periaqueductal gray
PaS: Parasubiculum
PaT: Paraventricular thalamic nucleus (=PV)
PCo: Posterior cortical amygdaloid nucleus (=CoP, ~PMCo
PH: Posterior hypothalamic nucleus
PIL: Posterior intralaminar thalamic nucleus
Pir: Piriform cortex
PLH: Peduncular part of lateral hypothalamus
PLCo: Posterolateral cortical amygdaloid nucleus (~PAC)
PMCo: Posteromedial cortical amygdaloid nucleus (~PCo)
PMv: Premammillary nucleus, ventral part
PP: Peripeduncular nucleus
PRh: Perirhinal cortex
PrL: Prelimbic cortex
ProSv: Prosubiculum, ventral part
PT: Paratenial thalamic nucleus
Re: Reuniens thalamic nucleus
rs: Rhinal sulcus
SLEA: Sublenticular extended amygdala (~EA)
SubG: Subgeniculate nucleus
SUM: Supra-mammillary nucleus
TeA: Temporal association cortex
Tu: Olfactory tubercle
VDB: Nucleus of the diagonal band, vertical limb
VEn: Ventral endopiriform nucleus
VIEnt: Ventral intermediate entorhinal cortex
VMH: Ventromedial hypothalamic nucleus
VP: Ventral pallidum
VTA: Ventral tegmental area
