# Supplementary figures and images for: Brain-wide connections of the parvicellular subdivision of the basolateral and basomedial amygdaloid nuclei in the rats
Source: Front Neural Circuits. 2025 Apr 25;19:1575232. doi: 10.3389/fncir.2025.1575232 (PMC12061975; doi:10.3389/fncir.2025.1575232)

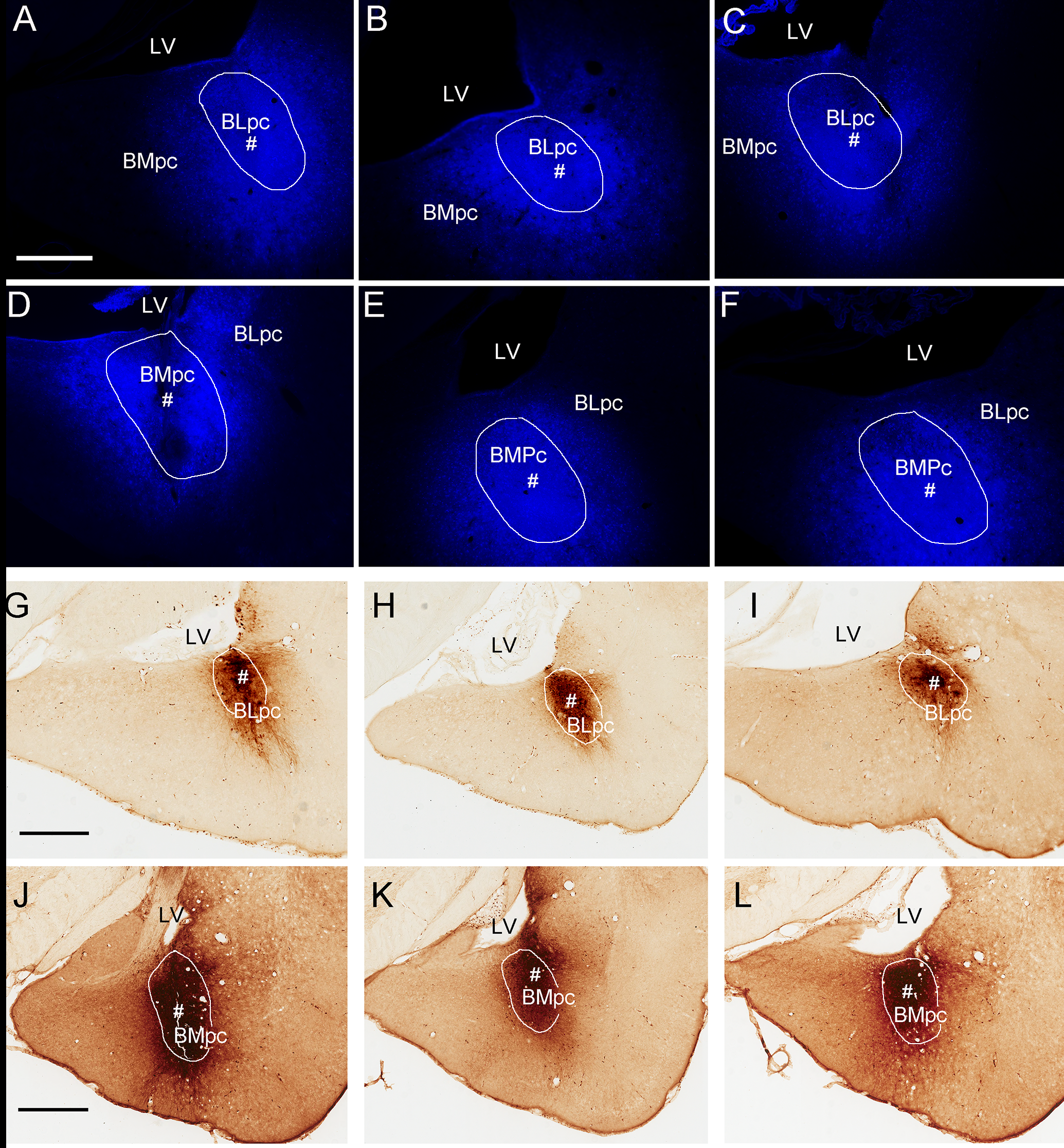

Supplement: SUPPLEMENTARY FIGURE 1 — Representative injection sites of the tracers FG and BDA. (A–F) Six FG injection sites in the BLpc (A–C) and BMpc (D–F). (G–L) Six BDA injection sites in the BLpc (G–I) and BMpc (J-L). The injection cores are indicated by #. Scale bars: 500 μm in (A) for (A–F); 600 μm in (G) for (G–I); 800 μm in (J) for (J–L). [file Image_1.tif]

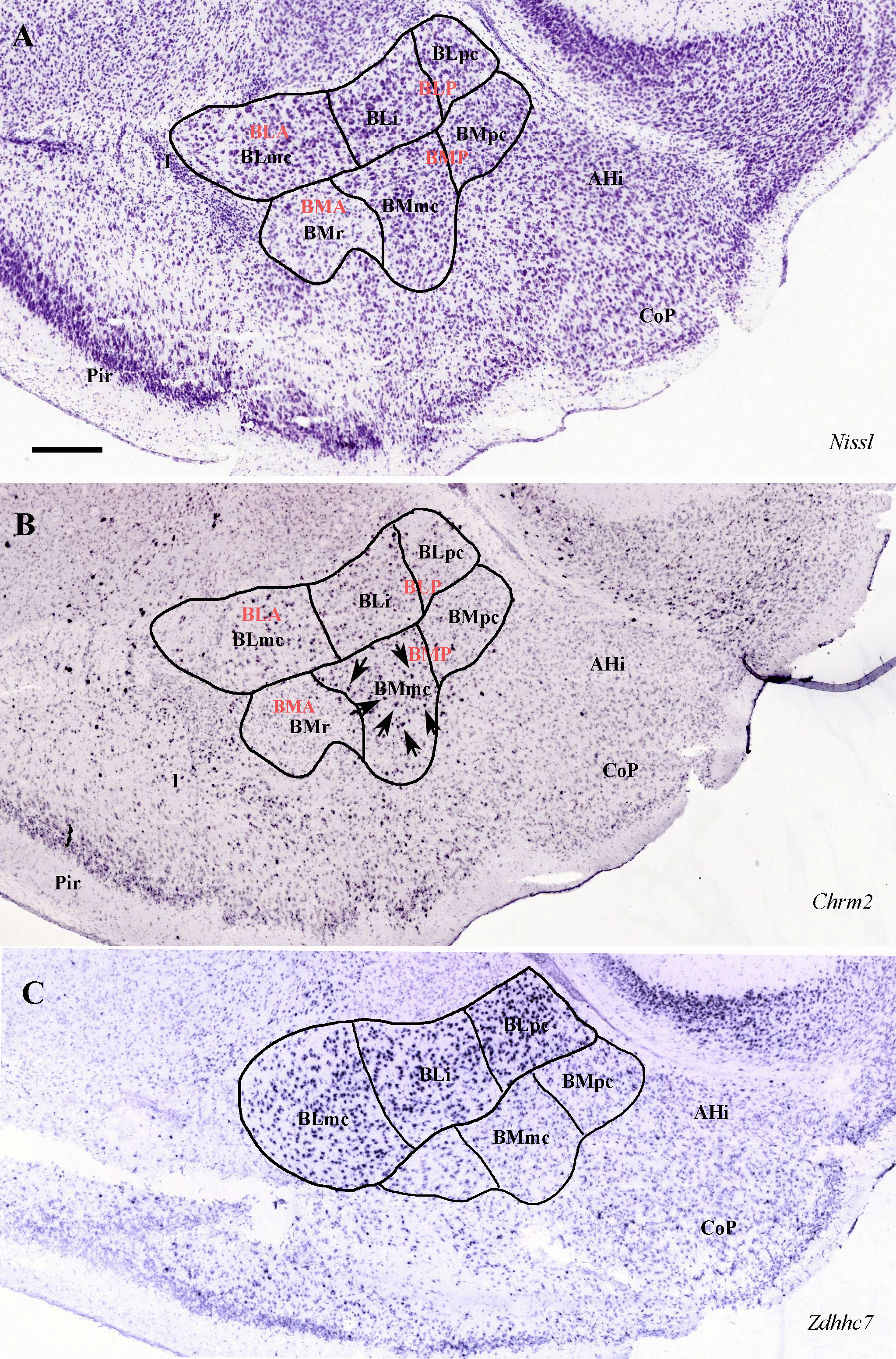

Supplement: SUPPLEMENTARY FIGURE 2 — Location, cytoarchitecture and subdivisions of the BL and BM in the sagittal sections of mouse brain. (A,B) Two adjacent sections stained for Nissl substance (A) and Chrm2 expression (B) showing the overall cell size (A) and Chrm2 expression (B) in the BL and BM subdivisions. The anterior and posterior subdivisions in orange color indicate those in most brain atlases while those in black color indicate the terms used in the present study. Note that BLmc and BLi display stronger Chrm2 expression signals than BLpc does. In contrast, BMr (BMA) and BMpc show faint Chrm2 expression whereas BMmc has stronger expression (indicated by arrows). (C) One closely matched sagittal section showing the strong Zdhhc7 expression in the BLmc, BLi and BLpc (with a gradient). In contrast, all BM subdivisions display faint expressions. This expression pattern makes the BL and BM stand out. Scale bar: 300 μm in (A) (for panels A–C). [file Image_2.tif]

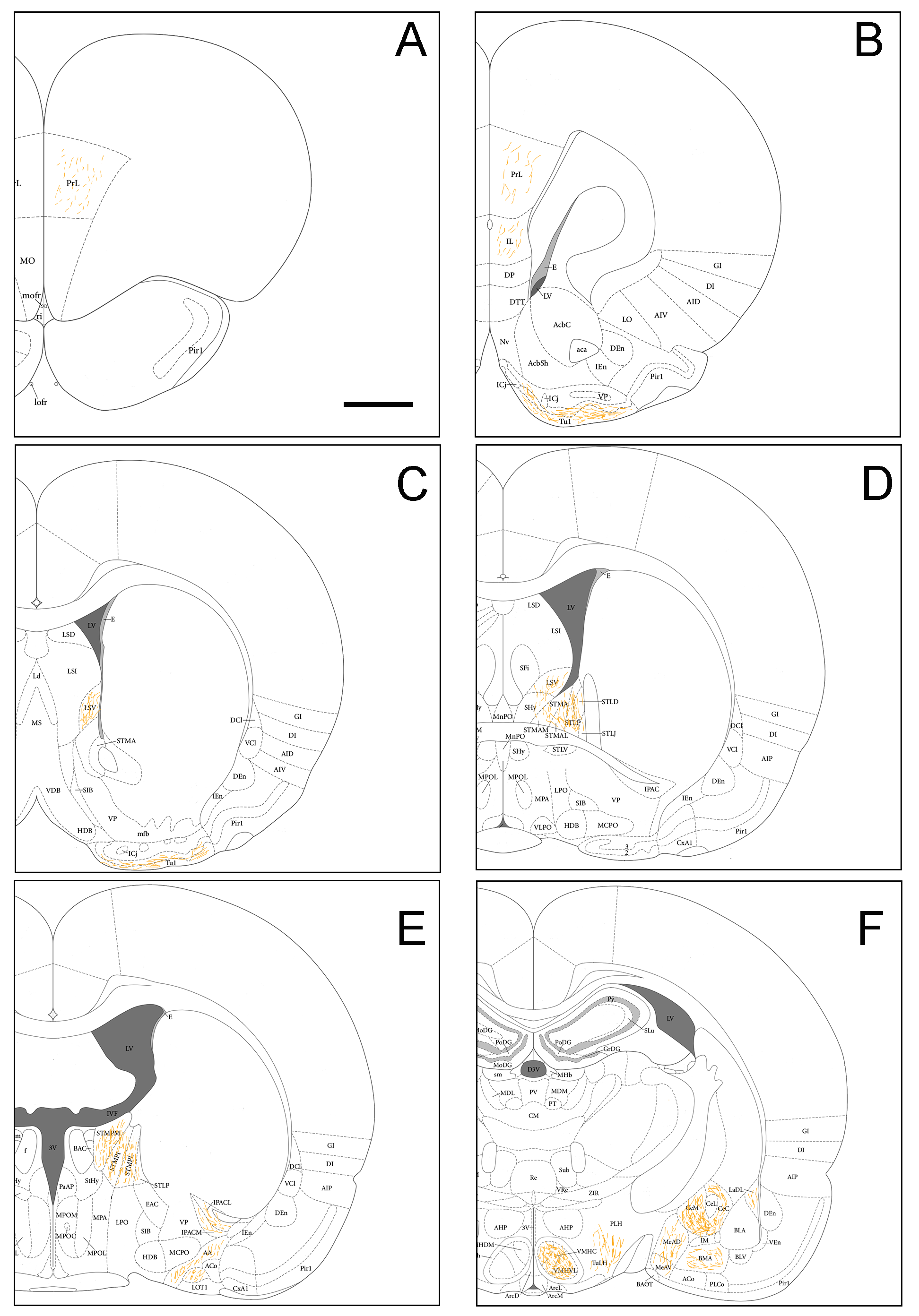

Supplement: SUPPLEMENTARY FIGURE 3 — Brain-wide efferent projections of the BMpc in the rat. (A–L) Schematic illustration of BDA-labeled axon terminals in sequential anterior (A) to posterior (L) coronal sections following a BDA injection into the BMpc. The injection site (#) is shown as the crowded dot regions (#) in panels (H,I). See text for explanation. For abbreviations see the list. Scale bar: 2 mm (applies to all panels). [file Image_3.tif]

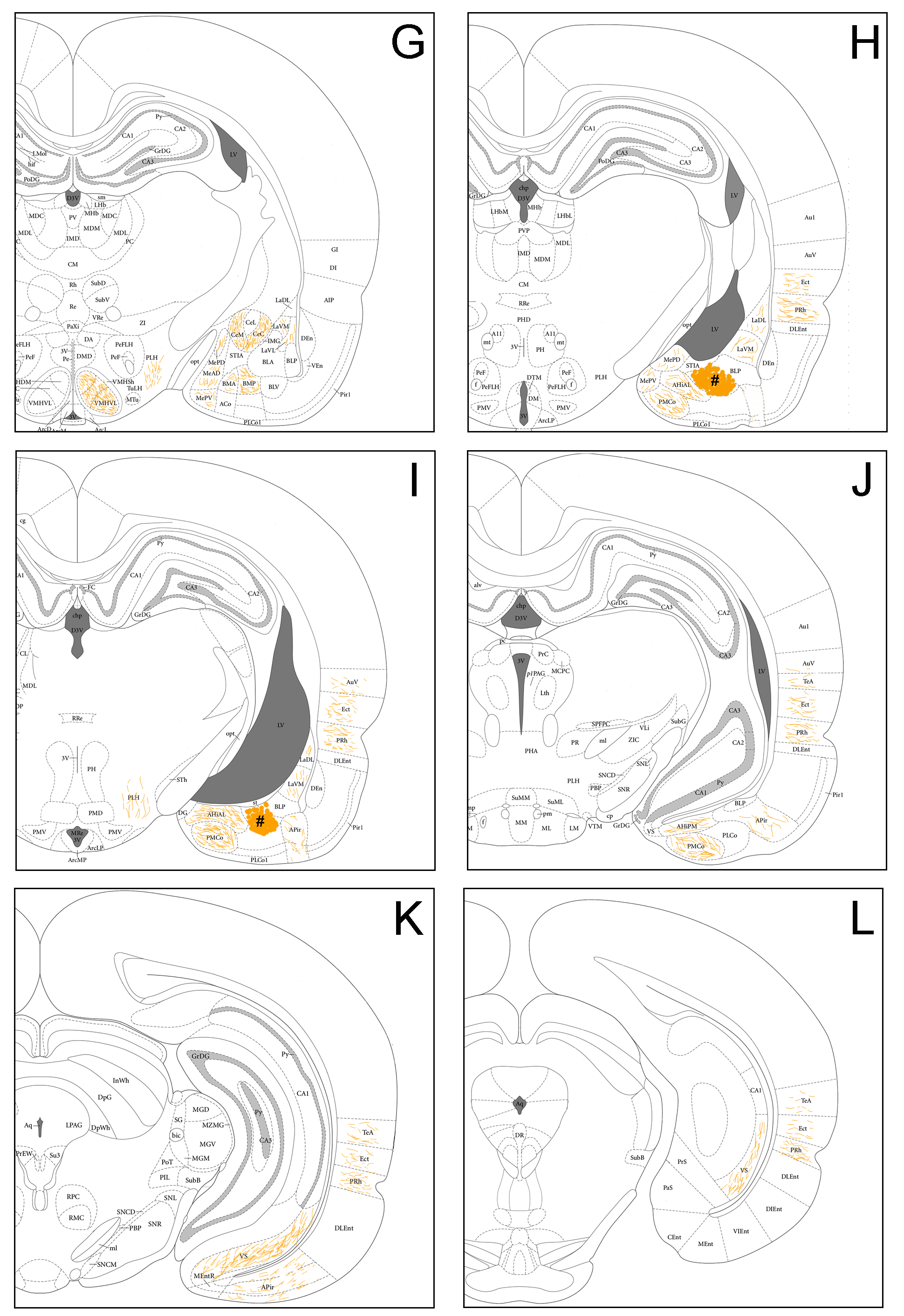

Supplement: Supplementary file 5 [file Image_4.tif]
